# Overcoming Thermal Degradation during Continuous Conversion of Water into Hydrogen Peroxide in a Flexible Plasma Reactor

**DOI:** 10.1021/jacs.5c14829

**Published:** 2026-03-02

**Authors:** Mery S. Hernandez, Yannis Mikolaiczyk, Sergey Soldatov, Guido Link, Lucas Silberer, Roland Dittmeyer, Alexander Navarrete

**Affiliations:** † Institute for Micro Process Engineering, 150232 Karlsruhe Institute of Technology, Karlsruhe 76344, Germany; ‡ Institute for Pulsed Power and Microwave Technology, 150232 Karlsruhe Institute of Technology, Karlsruhe 76344, Germany

## Abstract

By control of the nanosecond pulsation, energy input, and flow, it is possible to achieve commercial-level hydrogen peroxide (H_2_O_2_) concentrations using only water and plasma in a continuous process while minimizing thermal degradation. Time-resolved ultrafast Optical Emission Spectroscopy was employed to observe the formation of reactive species, shedding light on the underlying mechanisms. This study also found that thermal degradation has a critical role, which was effectively managed through quenching of the plasma zone. A parametric scan of pulse duration and pulse repetition frequency of the microwave power showed a significant influence on H_2_O_2_ formation, whereby the mean power also plays an important role. Additionally, the H_2_O_2_ concentration was found to be inversely proportional to the water flow rate. A maximum concentration of 0.17 wt % was achieved with 1.2 g/kWh based on the absorbed power at a flow rate of 0.2 mL/min. This plasma reactor technology shows promise for further development as a decentralized solution for the green chemical synthesis of H_2_O_2_.

## Graphical abstract

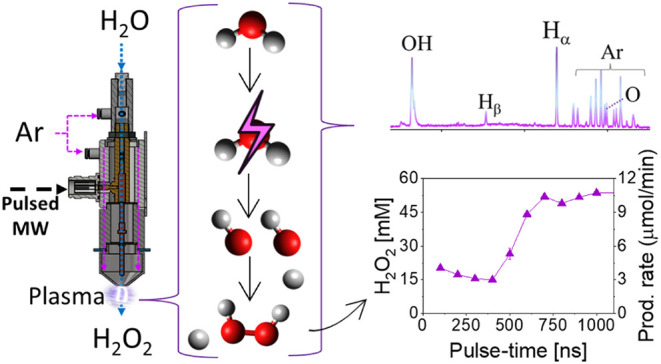

## Introduction

The demand for hydrogen peroxide (H_2_O_2_), a versatile and environmentally friendly oxidant, is rapidly increasing (4% per year).^1–3^ It is valued for its green properties, as it decomposes only into H_2_O and O_2_. Nevertheless, its traditional synthesis method, the anthraquinone oxidation process (AOP), relies on fossil feedstock and has significant drawbacks, including high carbon emissions, substantial waste generation, and challenges in transportation.^4,5^ These transportation challenges are related to the temperature-sensitive nature of H_2_O_2_; moreover, if it is highly concentrated, it is classified as a flammable liquid. A promising solution is the decentralized, on-site synthesis of H_2_O_2_ using renewable energy. This approach not only reduces the environmental impact of H_2_O_2_ synthesis but also supports the transition to renewable energy in the economy.^6^ Thus, as sustainability becomes a global priority, interest in greener alternatives for H_2_O_2_ production continues to grow.^7,8^ Several approaches have been explored as potential, sustainable options to the conventional AOP, including direct synthesis, photocatalysis, electrolysis, and plasma-based methods.^9–11^ Plasma technology potential lies on its rapid reaction rates, critical-materials-free operation, and compatibility with intermittent renewable energy sources, making them ideal for decentralized, on-site H_2_O_2_ production.^12,13^ High temperatures are detrimental for H_2_O_2_ synthesis;^14,15^ therefore, nonequilibrium cold plasma configurations (e.g., DBD) favoring lower temperatures have traditionally been used to increase the yield of H_2_O_2_.^13,16–23^ Microwave plasmas are not usually employed for hydrogen peroxide synthesis, mainly due to the high temperatures they can achieve (∼5–8 × 10^3^ K).^24^ Yet, in terms of efficiency, microwave (MW), warm plasmas have demonstrated high performance in other processes in which reaching high temperatures is not an issue.^25,26^ At the same time, for the plasma based synthesis of H_2_O_2_, it is necessary to first dissociate the water molecules and create OH radicals, which is favored by high temperatures (Figure S1). According to the literature, these radicals can then recombine to form H_2_O_2_.^27–29^ This last stage is a three-body reaction that is favored at lower temperatures due to increased collision efficiency.^30–33^ Looking to create a decentralized solution, we propose and demonstrate, for the first time, the use of a plasma reactor that makes use of nanosecond microwave pulsation to promote quenching of the plasma during continuous H_2_O_2_ synthesis at atmospheric pressure.^34,35^ The quenching promoted by ultrafast pulsation is complemented by the discharge contacting a cold surface provided by a heat exchanger (Figure 1a). Our results show near-commercial concentrations of H_2_O_2_ directly from water, without the use of catalysts or additional additives into the water, using a compact and tunable reaction system (Figure 1b).

**Figure 1:**
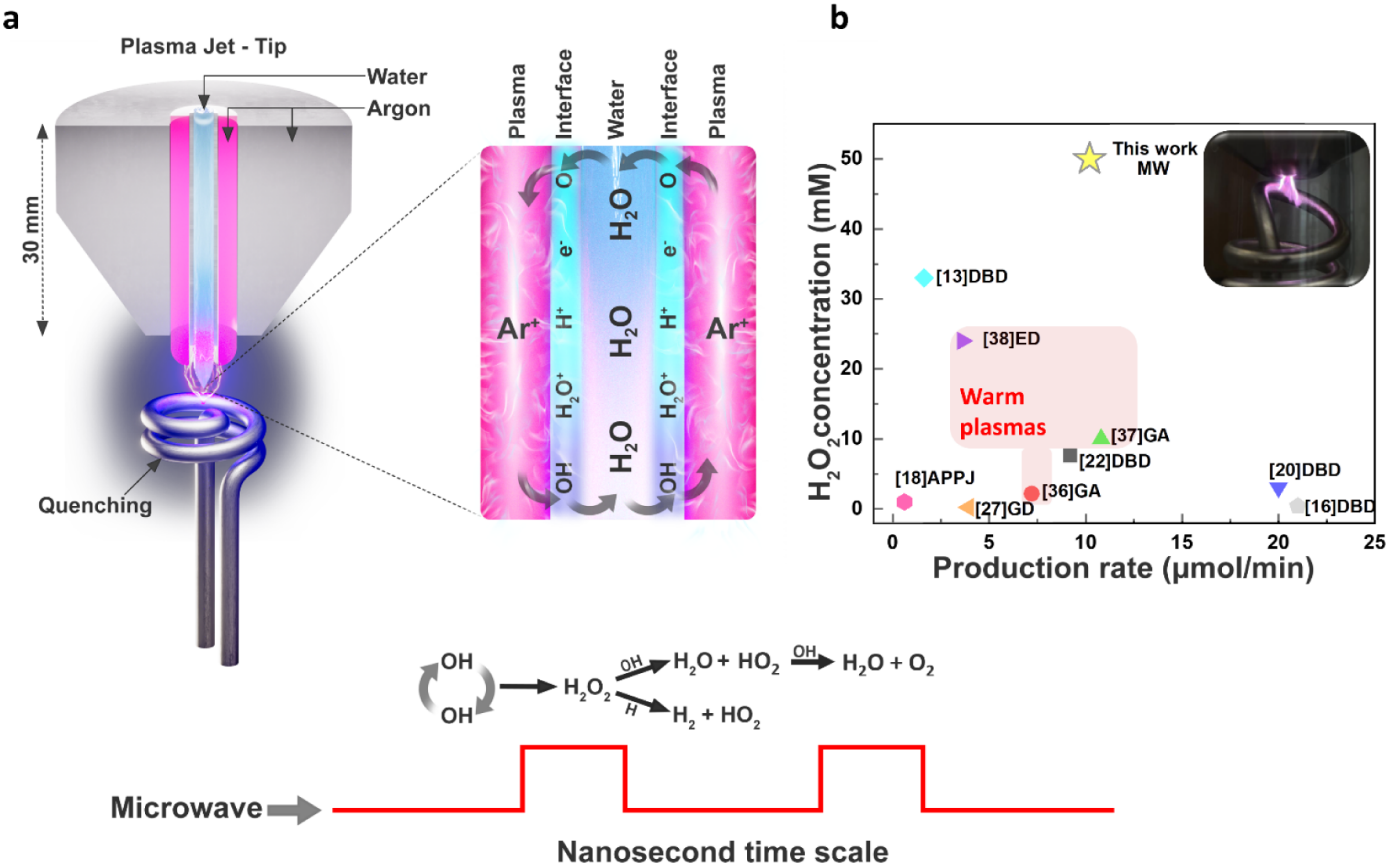
Nanosecond pulsed microwave plasma-based synthesis of H_2_O_2_ integrated with plasma quenching. (a) Schematics of the plasma at the tip of the reactor and the quenching system. (b) Comparison of the maximum H_2_O_2_ concentration achieved by this work and different plasma-based methods: Dielectric Barrier Discharge (DBD) using He+H_2_O,^13^ DBD using Ar+H_2_O vapor,^16^ Atmospheric Pressure Plasma Jet (APPJ) using He+H_2_O,^18^ DBD using NaCl solution added to H_2_O,^20^ DBD using H_2_O steam,^22^ Glow Discharge (GD) using He+H_2_O,^27^ Gliding Arc (GA) using Ar + H_2_O,^36^ GA using Ar+H_2_O spray,^37^ Electrical Discharge (ED) using Ar+H_2_O,^38^ and this work with Microwaves (MW) and Ar+H_2_O at the highest H_2_O_2_ concentration achieved within the study.

## Results and Discussion

The integration of the nanosecond-pulsed microwave plasma system with a cooling loop (using liquid water at 18 °C) (see Figure S2) for enhanced thermal quenching resulted in higher hydrogen peroxide (H_2_O_2_) recoveries (more details in Figure S3). This finding suggests that maintaining lower temperatures within the plasma–liquid interface zone is crucial for optimizing the H_2_O_2_ formation pathways and ensuring further stabilization. For a broad understanding of the microwave plasma system that we present here, a series of parametric studies were carried out with the plasma quenching included.

The H_2_O_2_ concentration was followed by UV–vis spectroscopy (more details in SI, Section 1). The duration of microwave pulses (pulse time or “t_on”) was varied within 100 ns and 10 μs, and the peak power was varied within 300 and 900 W. The duty cycle (DC) was calculated as the fraction of t_on over the period of pulsation (t_on + t_off), more details in Figure S4. The results of a scan of peak power along with pulse time at a fixed duty cycle of 0.3 and a water flow rate of 2.5 mL/min are presented in Figure 2a. Between 500 and 1000 ns, the H_2_O_2_ concentrations varied strongly across the applied peak powers, whereas other pulse time regions showed a more consistent trend. Specifically, at 700 ns of pulse time, corresponding to a pulsation frequency of 0.4 MHz (calculated as DC/t_on), the impact of peak power on H_2_O_2_ concentration was more clear as compared to 500 or 1000 ns of pulse time. At this specific pulse time (700 ns pulse time), H_2_O_2_ concentrations peaked at 6.5 mM and dropped to as low as 0.5 mM, along with peak power increase.

**Figure 2:**
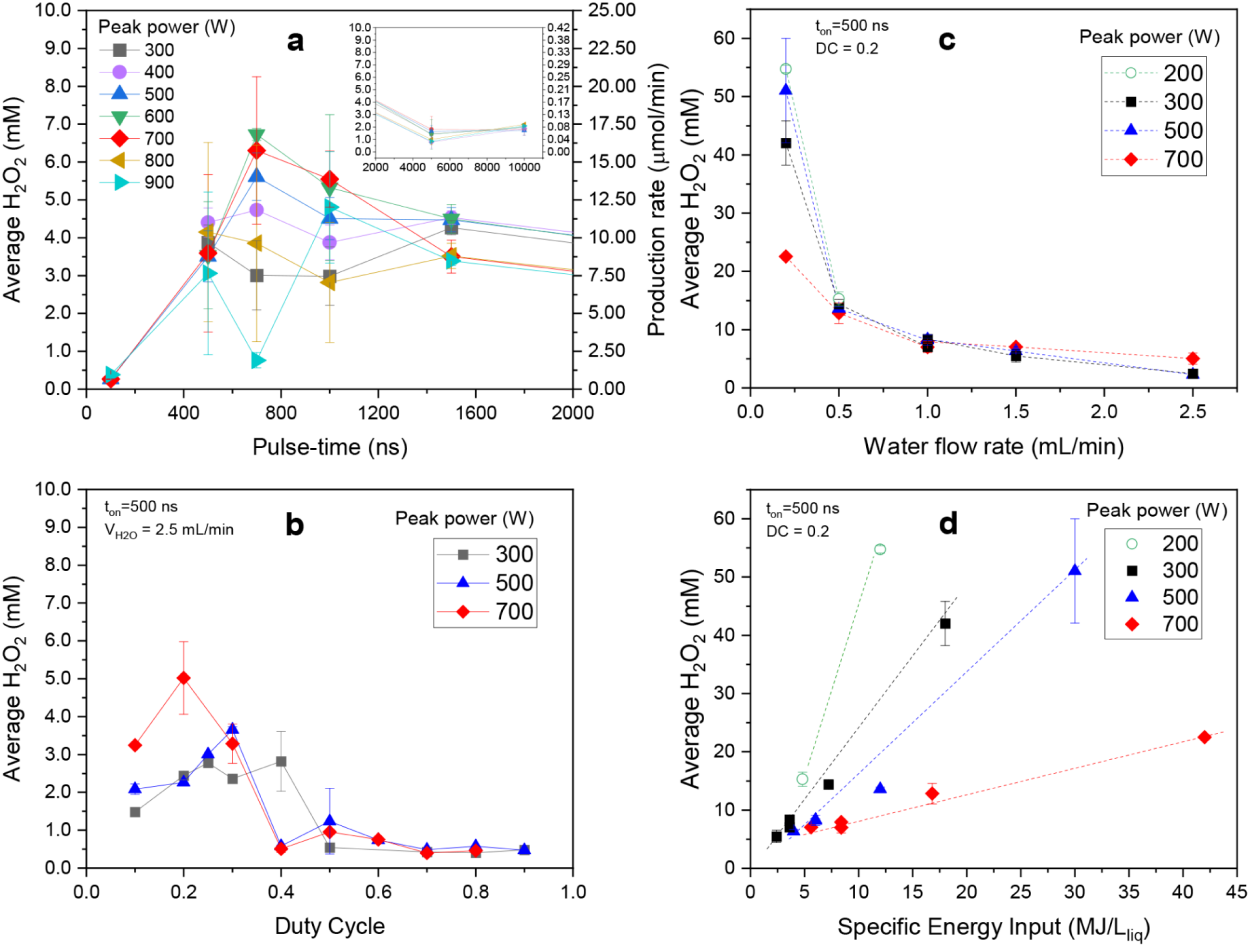
Experimental characterization of the system for H_2_O_2_ concentration in function of: (a) pulse time, (b) duty cycle, (c) water flow rate, and (d) specific energy input for different input power values. For figure (a), a constant water flow of 2.5 mL/min and a duty cycle of 0.3 were used. The duty cycle scan (b) is presented for a 500 ns pulse time and 2.5 mL/min of water. The water flow rate (c) and specific energy input per liquid flow (MJ/L liquid) (d) scans were taken at 0.2 DC and 500 ns of pulse time. The error bars in the graphics include three replicates. All experiments used 8.7 L/min of argon.

The results indicate that at a pulse time of 700 ns (at the specified conditions of flow and pulse frequency), the relationship between peak power and H_2_O_2_ concentration becomes more predictable. Coincidentally, the lifetime of certain short-lived reactive species involved in OH and H_2_O_2_ formation, such as the OH radical in its excited state, OH­(A), has an approximated lifetime of 700 ns.^21,39^ This coincidence simply highlights the importance of the pulse duration matching the characteristic lifetimes of some short-lived reactants; however, this does not imply that OH­(A) is the primary species for the overall H_2_O_2_ formation. Furthermore, for longer pulse-time values of 5 and 10 μs (see the inset in Figure 2a), concentrations lower than 3 mM were observed, with minimal variation across different peak powers. This clearly suggests that prolonged pulse times hinder H_2_O_2_ generation, which agrees with the very low H_2_O_2_ yield in continuous (DC = 1) microwave operation (Figure S5a). The most plausible explanation is the extended exposure of the active precursor species (e.g., the ground-state OH radical) and the newly formed H_2_O_2_ product to elevated gas temperatures. This prolonged thermal exposure promotes thermal degradation (dissociation or decomposition) of H_2_O_2_ lowering the overall observed concentration.^40^

As observed by Lietz et al. if the conversion of H_2_O into H_2_O_2_ (via electron impact dissociation to produce OH, followed by OH + OH + M → H_2_O_2_ + M) takes longer than the pulsation period, there would be an accumulation of OH from pulse to pulse, which could easily lead to H_2_O formation, or H_2_O_2_ consumption via OH + H_2_O_2_ → H_2_O + HO_2_.^40^ When a constant water flow of 2.5 mL/min was used, the average H_2_O_2_ production rate ranged from 0.6 to 15.6 μmol/min and varied proportionally with concentration.

In terms of energy yield (Figure S5b), values reached up to 0.20 g/kWh in average, affected mainly by the pulse time variation. One of our findings is that the use of continuous microwave power resulted only in low H_2_O_2_ concentrations, with a negligible variation along power input (Figure S5, line “CW”). Such finding supports the hypothesis that continuous microwaves tend to generate warm plasmas,^15^ which negatively impact the H_2_O_2_ production.^24^ Consequently, this reinforces the importance of using nanosecond pulsed microwaves to achieve lower gas temperatures, hence increasing the H_2_O_2_ production, given that H_2_O_2_ can be stabilized in water at low temperatures.^30^

By scanning peak power, we observed that it significantly affects the H_2_O_2_ production depending on the pulse duration. To explore this further, we chose three peak power settings that would give us an overview of the power effect: 300 W (low), 500 W (medium), and 700 W (high). We then proceeded to study the DC variation in steps of 0.1, from 0.1 up to 0.9. Varying the DC directly affects the mean power coupled into the plasma reaction zone (peak power × DC ≈ mean power). In this sense, higher duty cycles, which correspond to a higher mean power, should result in higher H_2_O_2_ production for the same peak power. In fact, Du et al. reported that both OH and H_2_O_2_ densities increased significantly with mean power input, with a factor of 2 times higher for argon than for helium gas, for a DBD plasma reactor at atmospheric pressure.^16^ Vasko et al. also reported a similar trend where higher duty cycles at a fixed peak power increased the concentration of H_2_O_2_ in an atmospheric pressure glow discharge reactor.^27^ Moreover, Soldatov et al. tested CO_2_ splitting in atmospheric microwave plasma at a fixed peak power of 220 W and found that the CO_2_ conversion increased with the DC, for different pulse durations.^34^ Wandell et al. observed a similar effect by studying H_2_O_2_ production in a pulsed water spray plasma reactor using a gliding arc: the production rate increased with the mean discharge power.^36^ However, a slight opposite trend was observed in the present work: lower concentrations were achieved along with increasing DC beyond 0.4, for every tested peak power (Figure 2b). Although increasing the duty cycle appeared to reduce H_2_O_2_ production, it is noteworthy that the highest H_2_O_2_ concentration was achieved at the highest tested peak power of 700 W, but at the lowest duty cycle of 0.2. In other words, at a pulse duration of 500 ns, a DC of 0.2 favors H_2_O_2_ formation under higher peak power conditions. The latter conditions correspond to a mean power of ∼140 W delivered in 500 ns bursts at a pulsation frequency of 0.4 MHz. Remarkably, the same frequency at which a peak concentration was previously found was at a fixed DC of 0.3 (Figure 2a). This suggests that optimum H_2_O_2_ concentrations can be achieved by using different microwave parameter combinations, particularly around a pulsation frequency of 0.4 MHz. Additionally, the DC scan at different peak power inputs revealed multiple H_2_O_2_ concentration peaks. For instance, a second peak was found for a DC of 0.4 at 300 W (Figure 2b), corresponding to 0.8 MHz and 120 W of mean power, while the other peak power inputs (500 W and 700 W) revealed rather high loss of H_2_O_2_ at such DC. Increasing the DC beyond 0.4 (Fq > 0.8 MHz) resulted in a decrease of H_2_O_2_ concentration, and for DC values between 0.7 and 0.9, variations in peak power had minimal effect. The latter observation could be related to an excess of thermal energy, which could negatively affect the formation reaction of H_2_O_2_ and its recovery from the plasma–water interface. Furthermore, the impact of the pulse duration was found to be less significant than that of the DC in determining H_2_O_2_ concentration (see Figure S6).

Notably, after analyzing the DC variation across different peak power inputs, the earlier observation of an optimum pulsation frequency around 0.4 MHz remains consistent. However, this optimum may shift under different water flow conditions. To explore this possibility, we next investigated the effect of varying water flow rates at a DC of 0.2, which previously yielded the highest H_2_O_2_ concentration. We selected similar peak power values as in the earlier tests (200, 300, 500, and 700 W), and the results are presented in Figure 2c. In general, decreasing the water flow rate had a positive effect on the H_2_O_2_ concentration, with a maximum average H_2_O_2_ of ca. 50 mM for 0.2 mL/min of water flow. This can be related to the increase of residence time at lower water flow rates allowing longer treatment of the water under pulsed plasma discharge. At 500 ns of pulse time and a DC of 0.2, the residence time of water in the plasma zone went from 508 ms (at 2.5 mL/min of water) to 6.36 s (at 0.2 mL/min of water), receiving ca. 2.5 × 10^6^ pulses of plasma in the latter case (more details in SI, Section 1). In the specific case of 0.2 mL/min of water flow, there was a clear difference between peak power variations, and the highest concentration was achieved at the lowest peak power applied of 200 W, while increasing the power resulted in a decreased H_2_O_2_ concentration. On the other hand, when examining H_2_O_2_ concentration as a function of water flow rate, each tested power input exhibited a consistent trend: the concentration decreased following a negative exponential relationship (Figure 2c). Such a trend appeared to be independent of both pulse-time or DC variations (Figure S7a-b). A similar behavior was reported by Cameli et al., who studied the effect of varying the liquid flow rate in a helical coaxial DBD plasma reactor. They found that H_2_O_2_ concentration decreased with increasing water flow rate, reaching a maximum of 33 mM at the lowest tested flow of 0.05 mL/min. The authors attributed this behavior to changes in the gas-to-liquid ratio, which influence droplet formation and the interfacial area available for plasma–liquid interaction.^13^ Such an explanation could also be applied in our case, given that, at a constant gas flow rate, lowering the water flow rate would only increase the residence time of the water and allow a longer exposure of water droplets to the plasma phase. Nevertheless, this does not explain why, at 0.2 mL/min of water, the maximum concentration was achieved at the minimum power input.

Looking at the specific energy input variation as a function of the water flow rate (SEI_L_), one can also have an additional parameter to analyze the plasma–liquid interaction. In our case, SEI_L_ variations between 5 and 44 MJ/L were observed for the different power inputs (Figure 2d). In general, higher SEI_L_ values (lower water flow rates) led to higher H_2_O_2_ concentrations at a fixed peak power, pulse time, and DC, following a quasi-linear trend represented by the dashed line Figure 2d. At the same time, the production rate ranged between 4.8 and 13.2 μmol/min, following apparent trends in which lower SEI_L_ values were associated with higher production rates (see Figure S7c-f). Additionally, to confirm the trend observed in Figure 2a, we performed a pulse time scan at a lower water flow rate of 0.2 mL/min (Figure S8), while maintaining a DC of 0.2. Here, we used peak powers of 300, 500, and 700 W. In this case, the H_2_O_2_ concentration shows a second rise between 500 and 1000 ns, like the behavior observed at 2.5 mL/min (Figure 2a). Peak concentration values reach ∼50 mM, matching earlier measurements (see Figure S8). These results confirm that reducing the flow rate enhances H_2_O_2_ production, while the pulse time influences concentration evolution.

The average energy yield (g/kWh) (calculated with the mean power, as explained earlier) was found within a range of 0.01–0.6 g/kWh, as presented in Figure 3. It is evident that some specific points present a higher performance (H_2_O_2_ concentrations >20 mM), as marked in the highlighted zone in the figure. These points belong to a water flow rate of 0.2 mL/min, which as discussed before led to the highest concentrations achieved here. Even though the trend is not linear, it is possible to tune the system aiming for higher energy yields without sacrificing the H_2_O_2_ concentration (more details in Figure S9).

**Figure 3:**
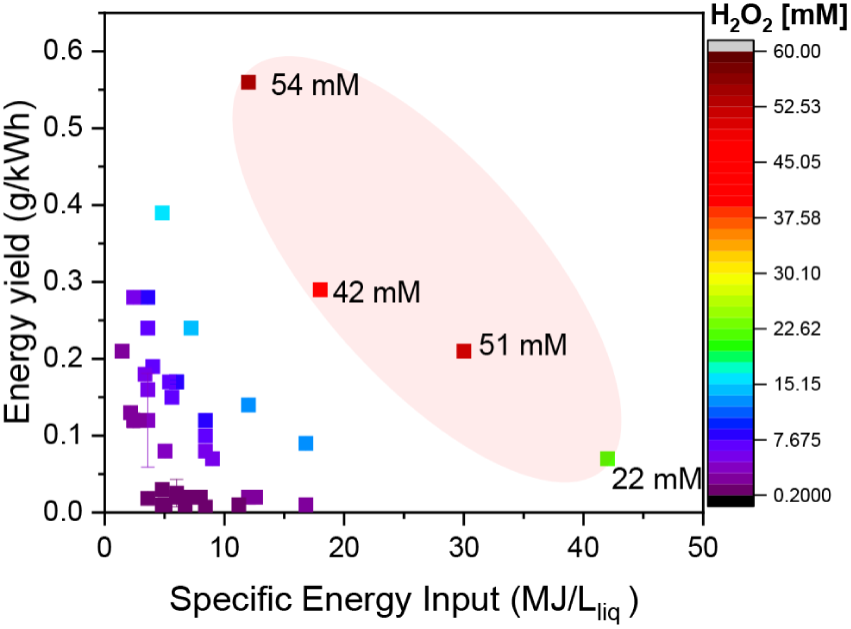
Energy yield versus specific energy input at different H_2_O_2_ concentrations. The results vary the water flow rate between 0.2 and 2.5 mL/min, the peak power between 200 and 700 W, and the duty cycle between 0.1 and 0.7, at a fixed pulse time of 500 ns. The highlighted area in the graphic indicates the energy yield of the highest H_2_O_2_ concentrations achieved while varying the specific energy input by adjusting the mean power at a water flow rate of 0.2 mL/min.

Nevertheless, after measuring the effective absorbed power (based on microwave energy absorbed by the plasma), we found that the energy yield was higher, reaching up to 1.2 g/kWh when yielding the highest H_2_O_2_ concentration (more details in Figure S10). The analysis of the energy usage and energy yield, as discussed in SI, Section 2, showed that a modification of the applied microwave power must be implemented to avoid high energy losses and increase the energetic efficiency.

The gas/liquid ratio was also analyzed for gas flow rates between 4 and 10 L/min and liquid flow rates of 0.2–5 mL/min. In general, higher H_2_O_2_ concentrations were found at larger gas-to-liquid ratios (>40 × 10^3^) within the studied ranges (more details in Figure S11).

The online use of Optical Emission Spectroscopy (OES) allows us to gather an initial qualitative assessment of the reaction mechanisms. For the specific case of 0.2 mL/min of water flow at 0.2 DC (which led to the maximum H_2_O_2_ concentrations here), a follow-up of the reactive species was obtained using a simple UV–vis spectrometer, as described in the experimental setup in SI, Section 1. The qualitative analysis is summarized in Figure 4, including temperature measurements of the gas near (2 cm away) the plasma–water interaction zone (temperature of the reaction environment) for tests without and with quenching. The behavior of the reactive species was evidently different for the experiments without quenching compared to those ones with quenching.

**Figure 4:**
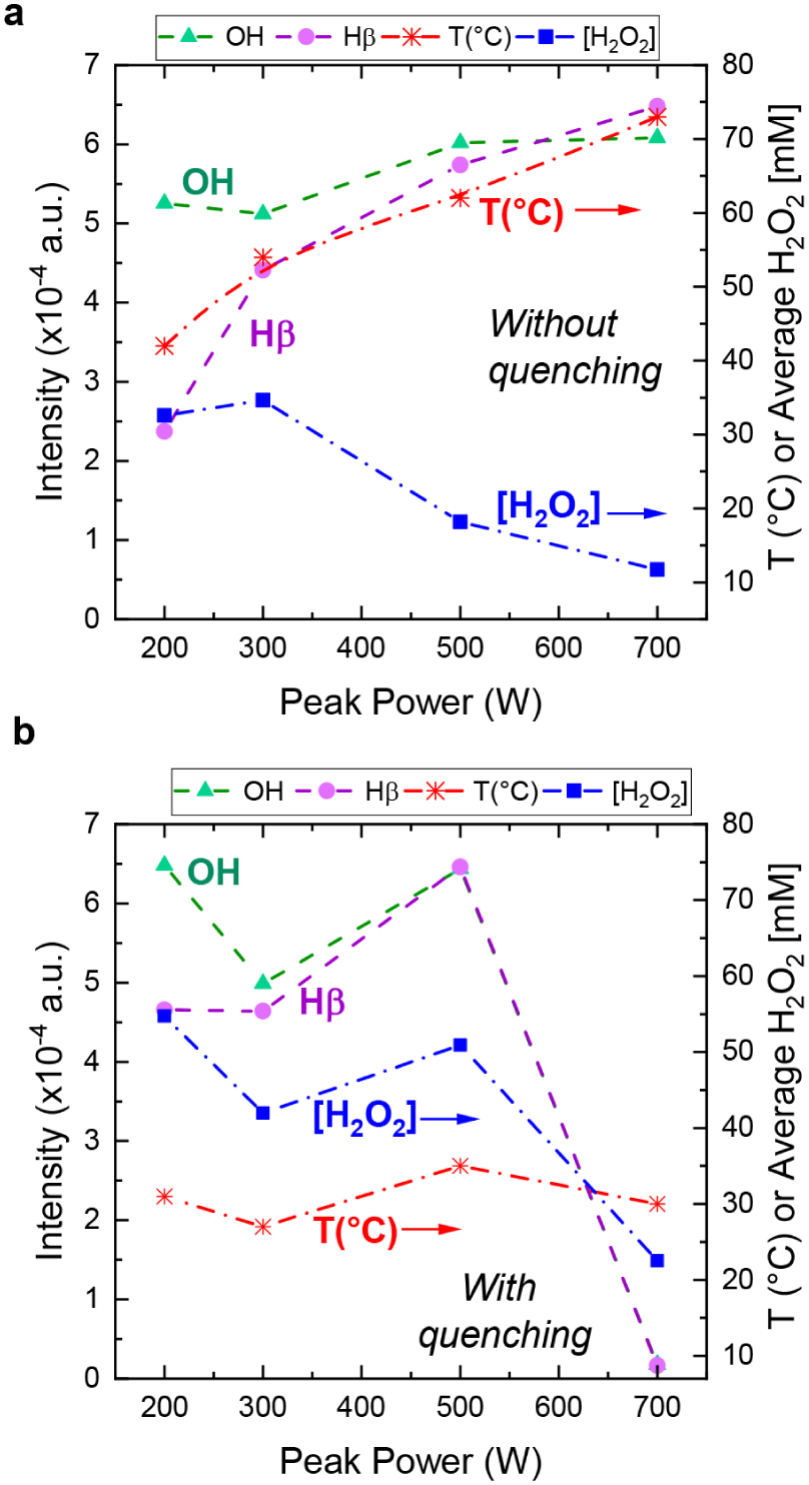
Qualitative spectral analysis of the emission intensity of species, gas temperature near the reaction environment, and H_2_O_2_ concentration as a function of the peak power. (a) Without quenching, (b) with quenching. The pulse time was fixed to 500 ns with a DC of 0.2 and 0.2 mL/min of water. Dashed lines are included in the graphic for visual guidance.

Before explaining the variation of the observed active species and the possible reaction mechanism, we can consider that the very first steps during plasma–water interaction are the water splitting and OH formation.^41^ The main reactions associated with the formation of OH (A-X) radicals via the dissociation of water molecules in argon plasma are electron impact dissociation (eq 1), electron–ion dissociative recombination (eq 2), dissociation by metastable species of argon (eq 3), Penning ionization for H_2_O^+^ production (eq 4), and thermal dissociation (eq 5) which occurs at elevated temperatures around 3500–4000 K, as reported before.^11,21,29,42^

1 $$e^{-}+H_{2}O\to OH+H+e^{-}$$

2 $$e^{-}+H_{2}O^{+}\to OH+H$$

3 $$Ar^{+}+H_{2}O\to OH+H+Ar$$

4 $$Ar^{*}+H_{2}O\to e^{-}+H_{2}O^{+}+Ar$$

5 $$H_{2}O^{*}+H_{2}O\to OH+H+H_{2}O$$

These reactions precede the formation of H_2_O_2_, due to ion recombination and radical interactions (eqs 6–12):^17,29^

6 $$OH+OH+M\to H_{2}O_{2}+M$$

7 $$HO_{2}+HO_{2}\to H_{2}O_{2}+O_{2}$$

8 $$HO_{2}^{-}+H^{+}\to H_{2}O_{2}$$

9 $$H_{3}O^{+}+HO_{2}^{-}\to H_{2}O_{2}+H_{2}O$$

10 $$HO_{2}^{-}+H_{2}O\to H_{2}O_{2}+OH^{-}$$

11 $$OH+H_{2}O^{*}\to H_{2}O_{2}+H$$

12 $$H_{2}O+H_{2}O\to H_{2}+H_{2}O_{2}$$

Eq 6 is a three-body reaction,^18^ where M, the collision partner, could be any atom or molecule, from Ar or H_2_O in this case. The formation of the HO_2_ and hydrated ion H_3_O species may originate from the following step reactions:^11,29^

13 $$2H_{2}O\to OH^{-}+H_{3}O^{+}$$

14 $$OH+O^{-}\to HO_{2}^{-}$$

15 $$H_{2}O^{+}\to OH+H_{3}O^{+}$$

Previous results from simulations have revealed that the OH radical species do no react with each other in the water phase,^43^ i.e., eq 6 takes place in the gas phase; however, the HO_2_ radicals could react with each other in the water phase to form H_2_O_2_ and O_2_ (eq 7).^43^ Nonetheless, the formation of H_2_O_2_ can take place in the gas, in the liquid, or at their interface. For the reaction at the interface, the formed H_2_O_2_ is then incorporated into the liquid.^44^ In general, the mechanisms occurring at the gas–liquid interface are very complex and still under investigation.^45^ It has been stated before that such reactions are dependent on the temperature conditions, which affect the collisions, adsorption, and absorption phenomena happening in the interface.^20^

High temperatures like those present in microwave plasmas are detrimental to the H_2_O_2_;^29^ therefore, not only the interaction of ions or radicals toward the formation of water (eqs 16 and 17) but also the H_2_O_2_ decomposition (eqs 18–20) due to thermal or radical or ion interactions need to be considered:^27,29^

16 $$OH+HO_{2}\to H_{2}O+O_{2}$$

17 $$OH+OH^{-}\to H_{2}O+O^{-}$$

18 $$OH+H_{2}O_{2}\to H_{2}O+HO_{2}$$

19 $$H+H_{2}O_{2}\to H_{2}+HO_{2}$$

$${e^{-}}_{aq}+H_{2}O_{2}\to OH^{-}+OH$$ 20

Having that in mind, we can observe that without plasma quenching (Figure 4a), the temperature in the afterglow rises along with microwave peak power. A consideration of the emission of excited states for OH radical (at 309 nm) and atomic hydrogen (H_β_ at 485 nm) can provide qualitative information on the relative formation trends of those species. The increasing trends of OH and H_β_ emission intensities versus peak power in Figure 4a may evidence most likely the enhancement of electron density or the rate coefficient for electron-impact excitation. The previous assumption is quite correlated with increasing peak power, while microwave power is directly transferred to electrons, which not only drive the ionization and excitation reactions but also promote the H_2_O dissociation through reactions 1–2. At the same time, the increase of peak power is leading to a decrease of the H_2_O_2_ concentration that suggests that not only the H_2_O_2_ formation pathways are taking place but also, and with more emphasis, the H_2_O_2_ decomposition pathways toward OH, O, O_2_ and H_2_O formation, favored by a rise in temperature, as represented in eqs 16–20. In the experiments with quenching, where the plasma flame gets in contact with the heat exchanger surface, the temperature was successfully decreased to around 30 °C (Figure 4b). This resulted in overall higher H_2_O_2_ concentrations, suggesting that H_2_O_2_ was kept alive due to the quenching effect. The intensities of excited H_β_ and OH were nonmonotonic and followed the trend of H_2_O_2_ concentration (Figure 4b). At a higher peak power of 700 W, a decline was observed in H_β_ and OH emission intensities, as well as in the resulting H_2_O_2_ concentration. A possible explanation for that is the overall quenching of the excited states at the metallic heat exchanger surface.

The nonmonotonic trend (with a peak at 500 W) can reflect the competition between excitation and de-excitation processes; however, a definitive interpretation is not possible based solely on the results of the OES measurements.

Complementary information can be obtained from the analysis of the gas composition. Therefore, a mass balance was calculated, along with selectivities to H_2_, O_2_ and H_2_O_2_, as the reaction products, and presented in SI, Tables S2 and S3. The analysis of the gas products revealed the presence of oxygen, hydrogen, and steam, which can come from eqs 7, 16–19, recombination reactions (eqs 21 and 22), or due to water splitting (eq 23).^29,33^ According to the gas composition, the process is more selective to H_2_ than to O_2_, possibly favoring the reaction in eq 19.

21 $$O+O\to O_{2}$$

22 $$H+H\to H_{2}$$

23 $$H_{2}O\to H_{2}+1/2O_{2}$$

With the information gathered so far, one can infer about the possible reaction mechanisms and determine species interaction toward the formation of H_2_O_2_. However, a temperature analysis of the plasma would clarify whether a thermal process is more dominant in this case.

The gas temperature can be determined following the rotational temperature distribution (*T*_rot_) of the OH (A-X) emission spectra.^41^ Qazi et al. discussed the different temperatures in an electrical discharge-generated plasma in contact with liquid, distinguishing between the gas and the liquid phase temperatures.^42^ Nevertheless, if the rotational distribution does not follow a Boltzmann distribution, then *T*_rot_ cannot represent the gas temperature correctly, and this is commonly observed for nonequilibrium plasmas.^46^ For instance, Bruggeman et al. studied a DC plasma discharge generated in water, investigating the discharge in the liquid mode and in the bubble mode. The authors reported OH rotational temperatures with a non-Boltzmann distribution around 2950 K, revealing that the efficiency of H_2_O_2_ formation is significantly smaller in the bubble mode than in the liquid mode.^47^

In this sense, the temperature in the plasma environment depends on the plasma system configuration. For an electrical discharge in water, a global reaction mechanism for H_2_O_2_ formation has been discussed, as described by eq 24.^44^

24 $$6H_{2}O\to 4H_{2}+O_{2}+2H_{2}O_{2}$$

Nevertheless, the plasma configuration and the way of contacting the plasma and the liquid medium highly affect the H_2_O_2_ formation paths. In this work, we calculated the reaction coefficients from the mass balance using the gas composition and liquid products, at a specific set of experimental conditions, resulting in the following expression:

25 $$27H_{2}O\to 26H_{2}+12.5O_{2}+H_{2}O_{2}$$

The mass balance details are presented in the SI, Table S1. The main difference between eqs 24 and 25 is the ratio between H_2_O and H_2_O_2_, having a molar ratio of ∼27:1 for the plasma configuration discussed here and a molar ratio of ∼27:26 between H_2_O and H_2_ (close to 1:1). The higher selectivity toward H_2_ and O_2_ formation rather than to H_2_O_2_ could be attributed to the dominance of OH-based reaction pathways to produce H_2_O_2_. As explained by Luo et al., the OH mainly produced by electron impact dissociation of water and hydration of H_2_O^+^ (eqs 1 and 15) follows a destruction through a 3-body radical–radical recombination with water as the third body.^48^ In electronegative (low-energy discharges) discharges, however, the recombination between positive ions (H_3_O^+^) and negative ions could significantly contribute to H formation, further leading to H_2_ formation by interaction with water molecules.^48^ If the production of H_2_O_2_ is dominated by radical recombination, the availability of energetic electrons becomes a key factor influencing the reaction pathway. In this context, the electron density can serve as a guide for the energy available in the system. Low electron densities (i.e., low-energy discharges) tend to favor ion-neutralization reactions and H_2_ formation, whereas high electron densities (or higher specific energy) promote OH radical generation through more frequent heavy-particle and electron-induced reactions, thereby enhancing H_2_O_2_ production.^48^ Nevertheless, the destruction of H_2_O_2_ is also caused by OH, H, and O species or by the amount of vapor.

Moreover, it has been reported that humidity can increase or decrease the level of formation of H_2_O_2_ under certain energy input conditions. Gorbanev et al. reported that at elevated humidity (i.e., >60% in the gas feed), the H_2_O_2_ in the liquid decreases.^41^ In our case, we did not observe a clear influence of steam generation on the H_2_O_2_ production rate (Figure S12). Nevertheless, we found that the system is more predictable at a DC of 0.2 while employing cooling, leaving room to correlate H_2_O_2_ and steam generation (see Figure S12).

To further explain these phenomena, we analyzed the temperature of the plasma zone following a high-resolution (HR) time-resolved OES, with 0.1 nm of resolution (Figure S13 presents the scheme of the configuration). The HR-OES results are summarized in Figure 5. First, a representative spectrum acquired with a low resolution of 2 nm is included in Figure 5a. The HR-OES for the OH (A-X) rovibrational spectrum is presented in Figure 5b. With spectra fitting in MassiveOES, we calculated the rotational temperature of OH species (*T*_rot_), and its variation along the pulse discharge is presented in Figure 5c. The spectra were recorded at different time delays between the OES acquisition and the beginning of the microwave pulse. A full span of time delay was 2533 ns, which covers a total pulse period of 2333 ns. In this case, the *T*_rot_ does not represent the gas temperature (non-Boltzmann distribution), given the nonequilibrium nature of this plasma configuration. Therefore, here *T*_rot_ is the temperature of the excited state and it is used as a guidance for possible reaction mechanisms. A typical *T*_rot_ uncertainty rounds to 50 K at a spectral resolution of 0.03 nm.^49^ In our case, the *T*_rot_ uncertainty was obtained from MassiveOES (error bars in Figure 5c) with maximum values around 170 K. The observed *T*_rot_ values were around 4000 K (Figure 5c) at the beginning of the pulse, which is higher than previous similar reports, indicating that the process starts following mainly a thermal dissociation.^29^

**Figure 5:**
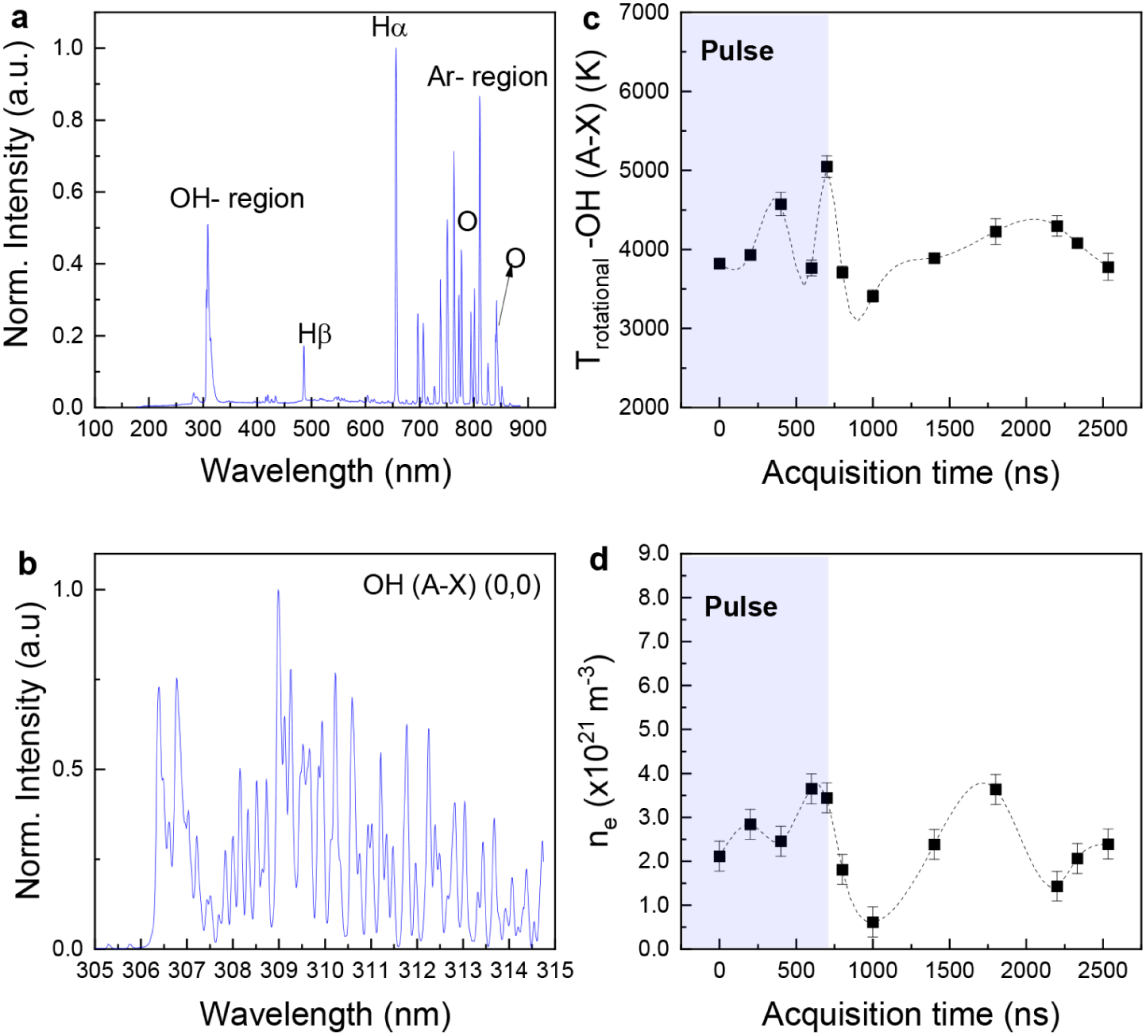
High-resolution OES. (a) Low-resolution overview of the OES spectrum, (b) high-resolution OES spectrum with characteristic OH rovibrational emission bands, (c) OH rotational temperature versus acquisition time, and (d) electron density versus acquisition time. The spectra were taken for 2.5 mL/min of water, 8.7 L/min of argon, 500 W of peak power, a pulse time of 700 ns, and an interpulse of 1633 ns (DC = 0.3), with a total pulse period of 2333 ns. In Figure 5b, the different lines indicate the OH transitions (A-X) for a representative acquisition time of 200 ns. The error bars in Figure 5c were taken from the MassiveOES fitting, and for Figure 5d, they represent the maximum *n*_*e*_ calculated using the spectral resolution of 0.1 nm as the fwhm.

The most prominent temperature (>5000 K) occurred at 700 ns. A decrease in temperature to below 3500 K was observed before 1000 ns, followed by a slight increase between 1400 and 2200 ns. Since the microwave pulse duration is 700 ns, this temperature evolution occurred during the interpulse period. During the microwave pulse, the electron impact (eqs 1 and 2) and thermal dissociation (eq 4) reactions are the main mechanisms for the formation of OH radicals. In this phase, the rotational energy is efficiently transported via vibrational-to-rotational (V-R) energy transfer, as energized electrons first excite vibrational modes, which subsequently relax into rotational excitation: *T*_e_ → *T*_vib_→ *T*_rot_.^30^ The efficiency and rate of V-R energy transfer vary depending on the collisional partners.

The increase in *T*_rot_ during the interpulse phase (Figure 5c) suggests that secondary excitation processes might be active. Given the relatively slow V–R relaxation of O_2_ (100–1000 ns at 4000–5000 K),^50^ compared to faster species like H_2_,^200^ it is plausible that ion–molecule reactions involving O_2_^+^ and H_2_O, such as H_2_O + O­(^1^D) → 2OH,^51^ could lead to OH radicals carrying additional rotational energy, reflected as an increase in *T*_rot_. Further validation of this hypothesis requires time-resolved plasma diagnostics and kinetic modeling, which are beyond the scope of this study.

We can consider that the observed trend of the *T*_rot_ in Figure 5c is representative of the general process given that the residence time of the water in the plasma–water reaction zone, at these conditions of 2.5 mL/min and 700 ns of pulse time, would be 508 ms. At the given 700 ns of pulse time, it is enough time for 2.18 × 10^5^ cycles of pulses (more details in SI, Section 1). For the observed *T*_rot_ values, the main OH formation pathway associated could fit to thermal dissociation (eq 5), which reported gas temperatures between 2500–5000 K.^21^

Similar variations as in the *T*_rot_ were also observed in the electron density (*n*_*e*_) behavior (Figure 5d). *n_e_* was estimated using the fwhm of the H_β_ line (SI, Section 3), as proposed in other studies.^47,52^ Our estimated *n*_*e*_ values, on the order of 10^21^ m^3^, are in agreement with previous reports for similar plasma setups.^47^ Moreover, the evolution of *T*_rot_ and *n_e_* in the interpulse time (after 700 ns, plasma off) could be related to longer living species (>1 ms) like H_2_, H_2_O_2_, and O_2_, as observed by Luo et al.^48^ The authors reported similar rising and decaying trends during the interpulse. Nonstabilized H_2_O_2_ in the interpulse time can act as an acceptor of hydrogen atoms and hydroxyl radicals, leading to its thermal decomposition.^53^ These reactions are exothermic and can contribute to an increase in temperature. Herein, the importance is of rapidly stabilizing the formed H_2_O_2_ before its thermal decomposition happens, supporting the value of adding cooling to the plasma–water interaction zone. For instance, a positive effect of fast cooling of a nanosecond plasma was also reported by Chauvet et al. The authors claimed that cooling of the plasma, due to bubble expansion, reduces the efficiency of second-order reactions, for instance, toward O_2_ formation via OH dissociation into O and H, which would participate in the decomposition of H_2_O_2_.^31^

In general, a thermal dissociation path for OH production appears to be more consistent with the observed rotational temperatures, followed by electron impact dissociation of water and further three-body radical recombination (eq 6),^54^ enhanced at atmospheric pressure. To develop a broader understanding of the plasma–water interface, we proposed a global classification of the key reactions according to the phase in which they are likely to occur; namely, the gas phase, liquid phase, or plasma–liquid interface (see Table S4). Overall, reactions taking place in the gas phase are faster than in the liquid phase; therefore, for a simplified plug flow reactor model (PFR), we considered a constant supply of H_2_O_2_ into the liquid phase. The model was fitted at specific conditions of pulse time (500 ns and 0.2 DC) and variation of power and residence time (see Figure S14). The fitting shows that at input powers of 200, 300, and 500 W the decomposition or the decay of H_2_O_2_ is negligible and that a higher residence time of the liquid in the plasma reaction zone would benefit the H_2_O_2_ production, as it is also evident in Figure S14. On the contrary, at a power of 700 W, the decay increases with residence time with a reaction rate constant of 0.04 s^–1^. This is in line with the former discussion about the detrimental effect of higher temperatures and steam generation that occurs at higher powers. Currently, the system is limited to a minimum liquid flow rate of 0.2 mL/min, and in principle, allowing longer residence time of the liquid in the plasma zone would improve the H_2_O_2_ production.

The general performance of the reactor has been compared to other works that used mainly water and plasma to produce H_2_O_2_. Figure S15 includes such comparison observing production rate, energy yield, and concentration and also provides a reference of an electrochemical synthesis example for context. The results show that the main contribution of our system is a higher concentration output, leaving room for further optimization of production rate and energy yield. Furthermore, the advantage of the system due to its on–off functionality makes it quite flexible for adapting to a renewable energy source, which is in line with global energy transition goals.

The present experiments employed argon as a carrier gas at flow rates of 4–10 L/min, which would imply large gas consumption in larger-scale systems. In any practical implementation, however, the argon would be operated in a recirculation loop (90–99%), with only a small makeup stream to compensate for possible losses. From a techno-economic perspective, our present energy yield (up to ≈1.2 g·kWh^–1^) is still modest compared to the most efficient electrochemical routes but lies in the upper range of plasma-only systems that use only water and electricity (see Figure S15). At the same time, our reactor achieves commercially relevant concentrations (≈50–54 mM, 0.17 wt %) without any additives or critical-metal catalysts and operates continuously for several hours with a variation of ±5 mM (0.017 wt %) (see Figure S16). The concept therefore targets a different niche than the centralized anthraquinone process or complex electrochemical stacks: decentralized, on-demand production from water and electricity, using noncritical materials and modular reactors that can be parallelized and combined with argon recirculation.

In this study, we demonstrated the feasibility of employing a conventionally considered warm plasma for the synthesis of thermally sensitive H_2_O_2_. The strategy consisted of using a nanosecond-pulsed microwave plasma, together with thermal quenching, to enhance hydrogen peroxide production from water. A systematic test revealed tunable parameters, with pulsation frequency playing a key role (notably around 0.4 MHz). The species observed (like OH and H_b_) provided insights into the reaction steps. High-resolution, time-resolved studies helped us look closer into how the process evolves along the microwave pulse. This technology represents a sustainable pathway for electrified H_2_O_2_ production, with strong potential for on-site, on-demand applications. As revealed by this work, a system capable of providing a higher residence time for the liquid phase in the plasma zone would improve the production of H_2_O_2_.

## Supplementary Material



## Notes

Plasma–liquid reactor representation, plasma–liquid pictures, parameters and calculations, HR-OES analysis, calibration for H_2_O_2_ concentration measurements, effect of plasma quenching, energy yield, production rate and gas-to-liquid ratio variation analysis, mass balance (PDF)
