# Supplementary material for: Overcoming Thermal Degradation during Continuous Conversion of Water into Hydrogen Peroxide in a Flexible Plasma Reactor
Source: J Am Chem Soc. 2026 Mar 2;148(10):10378–87. doi: 10.1021/jacs.5c14829 (PMC13003438; doi:10.1021/jacs.5c14829)
Supplement: Supplementary file 1 [file ja5c14829_si_001.pdf]

Overcoming thermal degradation during continuous conversion of water into hydrogen peroxide in a flexible plasma reactor

Mery S. Hernandez*, Yannis Mikolaiczyk*, Sergey Soldatov~, Guido Link~, Lucas Silberer~, Roland Dittmeyer*, Alexander Navarrete[1]*

*Institute for Micro Process Engineering, Karlsruhe Institute of Technology, Karlsruhe, 76344, Germany

~Institute for Pulsed Power and Microwave Technology, Karlsruhe Institute of Technology, Karlsruhe, 76344, Germany

[1]*Email: alexander.navarrete@kit.edu

Supporting Information

Section 1: Methods

Simulation of chemical equilibrium

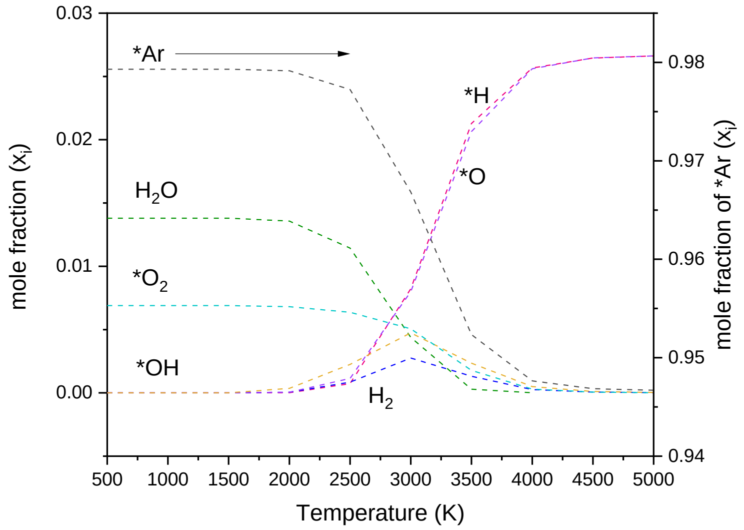

Figure S1. Influence of temperature on water dissociation. Simulation of chemical equilibrium of argon (98.8%) and H_2O using the CEA-NASA tool.(1) The OH species show a maximum generation at temperatures between 3000 and 3500 K. The condition studied was H_2O/argon mass ratios of 0.012 (water flow rate of 0.2 mL/min) at a constant argon flow of 8.7 L/min. The right y-axe reads the mole fraction of argon metastable species in the system.

Experimental Setup

The plasma reactor consists of a coaxial torch (max. 500 W, RF 4.310, Heuermann HF-Technik GmbH) connected to a solid-state microwave source (max. 1kW, TRUMPF Hüttinger Microwave) via a coaxial cable (Fairview 7/16 DIN). Argon gas is fed into the torch using mass flow controllers (MFC, Vögtlin), while liquid water is supplied thorugh a 1.8 mm (outside diameter) stainless steel tube connected to a water pump (1HM, Eldex). To monitor the temeperature of the reaction environment, a thermocouple is placed approximately 2 cm away from the plasma zone. An optic fiber connected to a UV-Vis (low resolution) detector (USB2000, Ocean Optics) allows real-time monitoring of species during the plasma-water interaction. Figure S2 provides a schematic representation of the experimental setup. All components (gas and liquid-dropwise) interact only at the tip of the torch, where the plasma formation occurs due to electromagnetic field concentration, as represented in Figure S3. The liquid product is collected downstream using a three-way valve, enabling specific sample collection as needed. To prevent H_2O_2 thermal decomposition, quenching of the plasma zone is attempted using a stainless steel loop (1/8") located below the plasma zone.

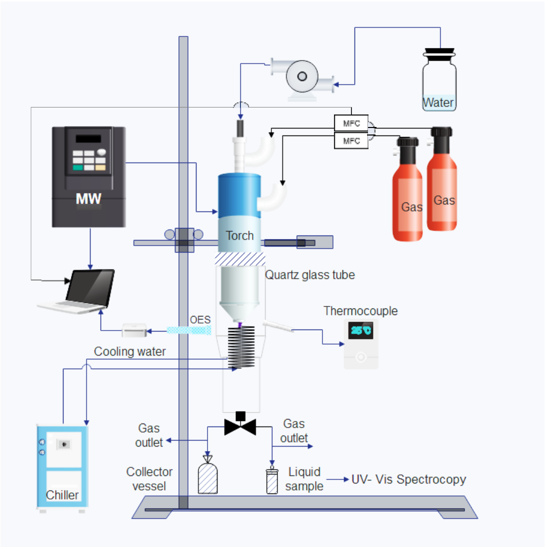

Figure S2. Representation of the plasma experimental set up. The gas flow was calculated in normal conditions using the vogtlin MFC control software *get read-y*. The microwave source was controlled via a Matlab GUI program developed at KIT.

Plasma quenching

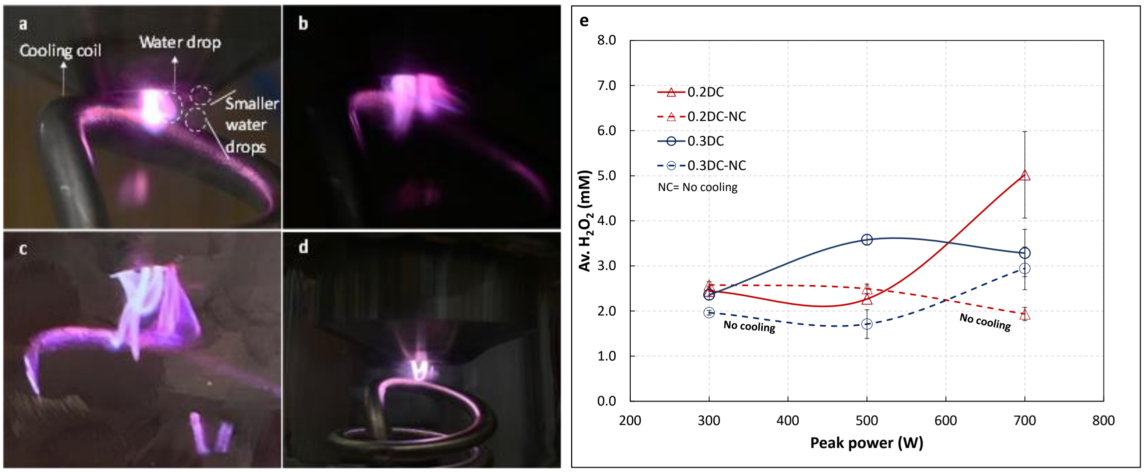

Figure S3. Effect of the cooling coil for plasma quenching. Pictures of plasma-water interaction and contact with the cooling coil surface (a-d), and effect of cooling over the H_2O_2 concentration (e). As circulating cooling liquid, deionized water at 18°C was used. For the experiments in (e), a fixed feed of water flow of 2.5 mL/min and a pulse time of 500 ns were compared with and without using the cooling coil (located below the plasma zone) for duty cycles of 0.2 and 0.3 at different power inputs (e). The dotted lines represent the experiments without cooling. The triangle marker represents a duty cycle of 0.2 and the circle marker represents the duty cycle of 0.3, and NC stands for no cooling in the experiments.

In Figure S3a, a photo of water droplet interaction with plasma shows how the water drops leaving the torch tip would encounter the plasma zone and break into countless smaller drops. The plasma-water interaction forces both phases to shake around the tip of the torch, as represented in Figure S3b. The variation of the plasma zone while interacting with the water phase, above the cooling coil can be seen in Figure S3c-d. For a peak power of 300 W, the effect of the cooling is less accentuated, as shown in Figure S3e, whereas for 500 W, adding the cooling coil represents an increment in H_2O_2 concentration of almost 2x for a DC of 0.3 (i.e. 6 MHz and ~150 W of incident power). This effect was visible also for 0.2 DC, but this time at 700 W (i.e. 4 MHz and 140 W of peak power), where increasing the power input seems to increase the H_2O_2 concentration while using quenching. In the opposite case (without quenching) it would result in a higher loss of H_2O_2 as shown in the figure for these conditions. In general, adding the quenching inverts the shape of the curve affecting positively the H_2O_2 production.

H_2O_2 concentration measurement

The H_2O_2 concentration in the plasma treated liquid was measured using the common titanium oxysulfate method. H_2O_2 can react with titanium sulfate in strong acid to form pertitanic acid (H_2TiO_4) (Eq.S1) and the absorption intensity of the yellow-coloured H_2TiO_4 at 407 nm is proportional to the reacted H_2O_2 concentration.(2) An illustration of the yellow coloured solutions reflecting the H_2O_2 concentration is presented in Scheme 1.

$$Ti^{4+} + H_2O_2 + 2H_2O \rightarrow H_2TiO_4 + 4H^+ \qquad \text{Eq. S1}$$

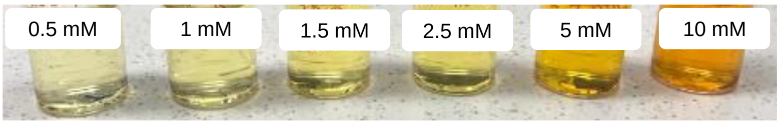

Scheme 1. Illustration of yellow colour variation of titrated samples, reflecting the H_2O_2 concentrations.

A titanium oxysulfate solution 0.16 M was prepared in the lab adding 25 g of titanium oxide sulfate ($TiOSO_4$ xH_2O + H_2SO_4, Thermoscientific) in 1 L of a H_2SO_4 solution 2 M. The final solution was kept in the fridge at 4°C. The calibration of the H_2O_2 was made using a 30% H_2O_2 commercial solution (Merck). Five dilution points were made in the milimolar range. For UV-Vis analysis, the 0.16M $TiOSO_4$ solution was added 1:1 to the prepared H_2O_2 solutions and finally diluted with 5 mL of water (deionized).(3) The calibration curve (Scheme 2) was obtained by plotting the intensity of absorbance against the known concentration. The concentration (c) of H_2O_2 can be determined using the Beer-Lambert Law (cuvette of 1 cm).

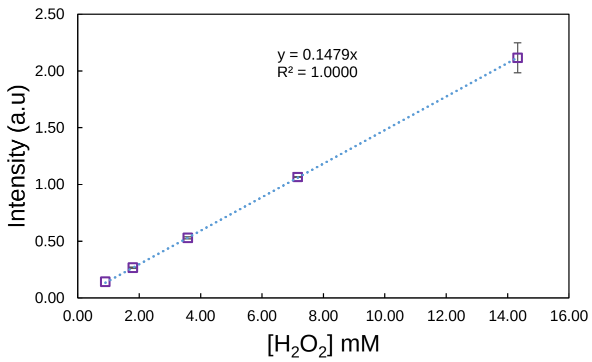

Scheme 2. Calibration curve for H_2O_2 analysis via UV-Vis Spectroscopy

Then the intensity divided by the calibration factor gives the concentration of H_2O_2 (I= c* 0.1479). The maximum intensity at 2 units presented higher deviation, therefore samples with concentrations higher than 12 mM were previously diluted with a factor of 11 to be able to read the absorbance.

Plasma parameters

The main studied parameters included the microwave peak power (W), time of active pulse (pulse time (ns)), duty cycle (DC), gas and liquid flow rates. The DC was calculated as the fraction of pulse time over the complete pulse period (Eq. S2 and Figure S4).

$$DC = \frac{t_{on}}{t_{on} + t_{off}} \quad \text{Eq. S2}$$

$$Frequency\ of\ pulsation\ (MHz) = \frac{DC}{t_{on}} * 1000 \quad \text{Eq. S3}$$

The production rate ($r_{product}$) of H_2O_2 was estimated in function of the H_2O_2 concentration and volumetric water flow (Equation S4).

$$r_{product}\ (\mu mol/s) = [H_2O_2] * \dot{V}_{H_2O} \quad \text{Eq. S4}$$

The specific energy input (SEI) was estimated in function of the of gas or liquid as follows:

$$SEI\ (kJ/L_{Ar\ or\ H_2O}) = \frac{(P * DC)}{\dot{V}_{Ar\ or\ H_2O}} \quad \text{Eq. S5}$$

Where P represents the peak power (W), and the power multiplied by the DC provides the effective energy input. The energy yield was estimated by Eq. S6.

$$E_{yield}\ (g/kW\ h) = \frac{r_{product} * M}{P * DC} \quad \text{Eq. S6}$$

To estimate the residence time (Eq. S7) of the gas (t_{gas}) in the plasma zone, the volume of gas discharge was considered as a cylinder with 3 mm diameter and 3 mm height. For the residence time of the liquid in the plasma zone (t_{liquid}) (Eq. S8), an estimated water drop size of 2.4 mm was considered. The number of discharge cycles can be estimated with Eq. S9.

$$t_{gas} = \frac{volume\ of\ discharge\ gap}{\dot{V}_{Ar}} \quad \text{Eq. S7}$$

$$t_{liquid} = \frac{volume\ of\ discharge\ gap}{\dot{V}_{H_2O}} \quad \text{Eq. S8}$$

$$discharge\ cycles\ in\ t_{gas\ or\ liquid} = \frac{t_{gas\ or\ liquid}}{t_{on} + t_{off}} \quad \text{Eq. S9}$$

For instance, at microwave conditions of 500ns t_on, 0.2 DC, 8.7 L/min of gas and 2.5 mL/min of water, the residence time of gas was ~ 0.15 ms and the water was ~508 ms, with a liquid plasma treatment of ~ 0.85 mL/min and a number of discharge cycles over the water volume of ~2.18×10^5. When changing the water flow to 0.2 mL/min keeping the same conditions as before, the residence time of the water increases to 6361.7 ms, and a number of discharge cycles over the water volume of ~2.5×10^6, while the residence time of the gas kept constant.

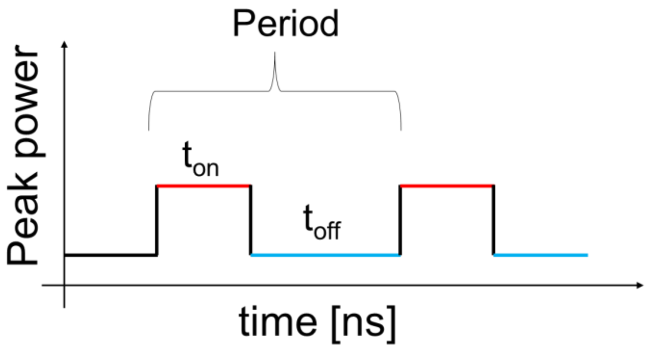

Figure S4. Pulsation period. The duty cycle (DC) was calculated by Equation S2. The sum of the pulse time (t_on) and inter-pulse time and (t_off) represents a complete pulse period and the repetition of pulses is called frequency of pulsation, which can be estimated with equation S3. Additionally, from the power and DC, it is possible to estimate the incident power into the system (peak power*DC ≈ mean power).

Section 2: Results

Scan of peak power input over H_2O_2 concentration and production rate.

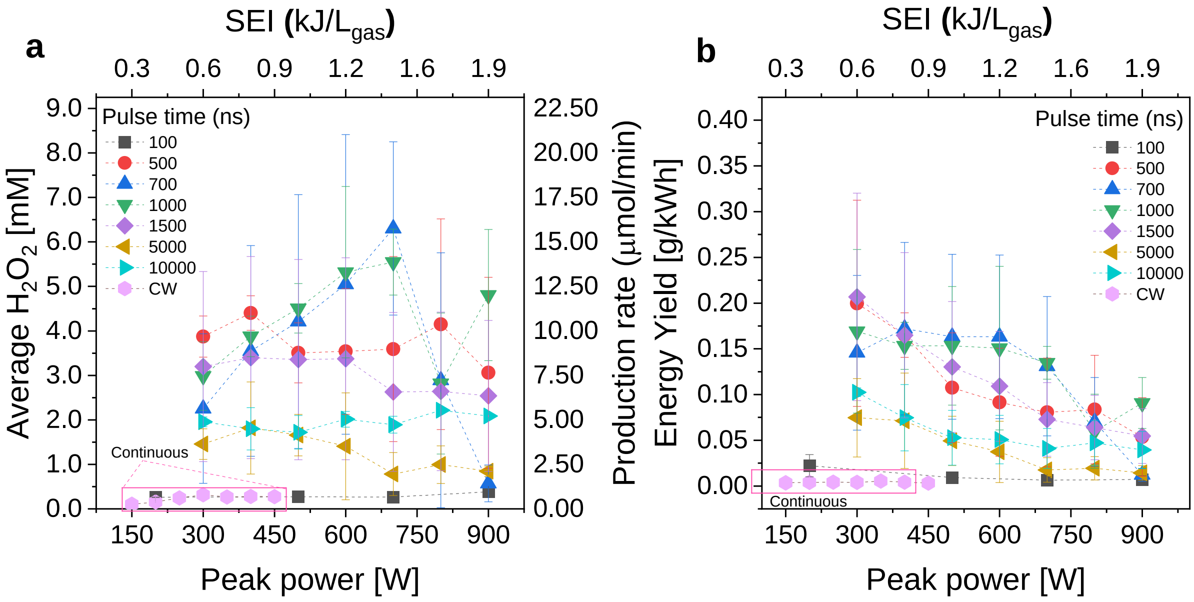

Figure S5. Average H_2O_2 concentration, production rate and Energy yield in function of the peak input power. The highlighted dots in the graphics represent the continuous microwave (CW) operation where only very low concentrations (up to 0.4 mM) and energy yields <0.01g/kWh were observed. The experiments were carried out at a fixed water flow of 2.5 mL/min, 8.7 L/min of argon, and a duty cycle of 0.3. In CW mode, the peak power axis corresponds to CW microwave power applied, and the difference to using nanosecond pulsations is quite evident. Only a pulsation time of 100 ns reached also low concentrations similar to the continuous mode, probably due to insufficient incident power at the given experimental conditions.

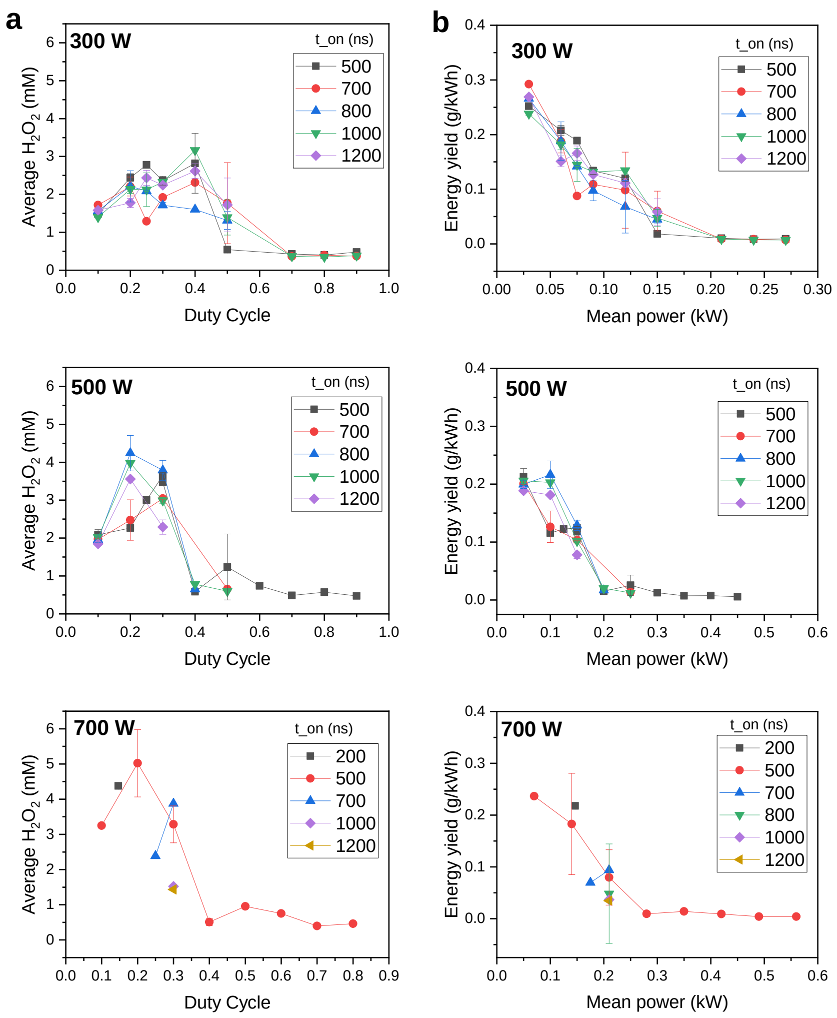

Figure S6. Analysis of: (a) duty cycle variation over H_2O_2 concentration (left column graphics) and (b) mean power over energy yield (right column graphics). For the experiments, three peak powers of 300, 500 and 700 W were studied at a liquid flow rate of 2.5 mL/min, and argon gas flow rate of 8.7 L/min. The graphic includes the variation of the pulsation time (500, 700, 800, 1000 and 1200 ns) and error bars for three replicates. In Figure S6a, the variation of pulse time has a higher effect at a DC of 0.2, while for the rest of DC values, the impact of pulse time was minimal among the different tested powers. This suggests that at a fixed liquid flow rate, the duty cycle (DC) would be the most influential parameter, followed by pulsation time, and then peak power. Furthermore, as illustrated in Figure S6b, varying the DC can be interpreted in terms of incident power. We observed that lower mean powers were associated with higher energy yields, and this relationship exhibited an almost linear negative slope for mean powers below 0.2 kW.

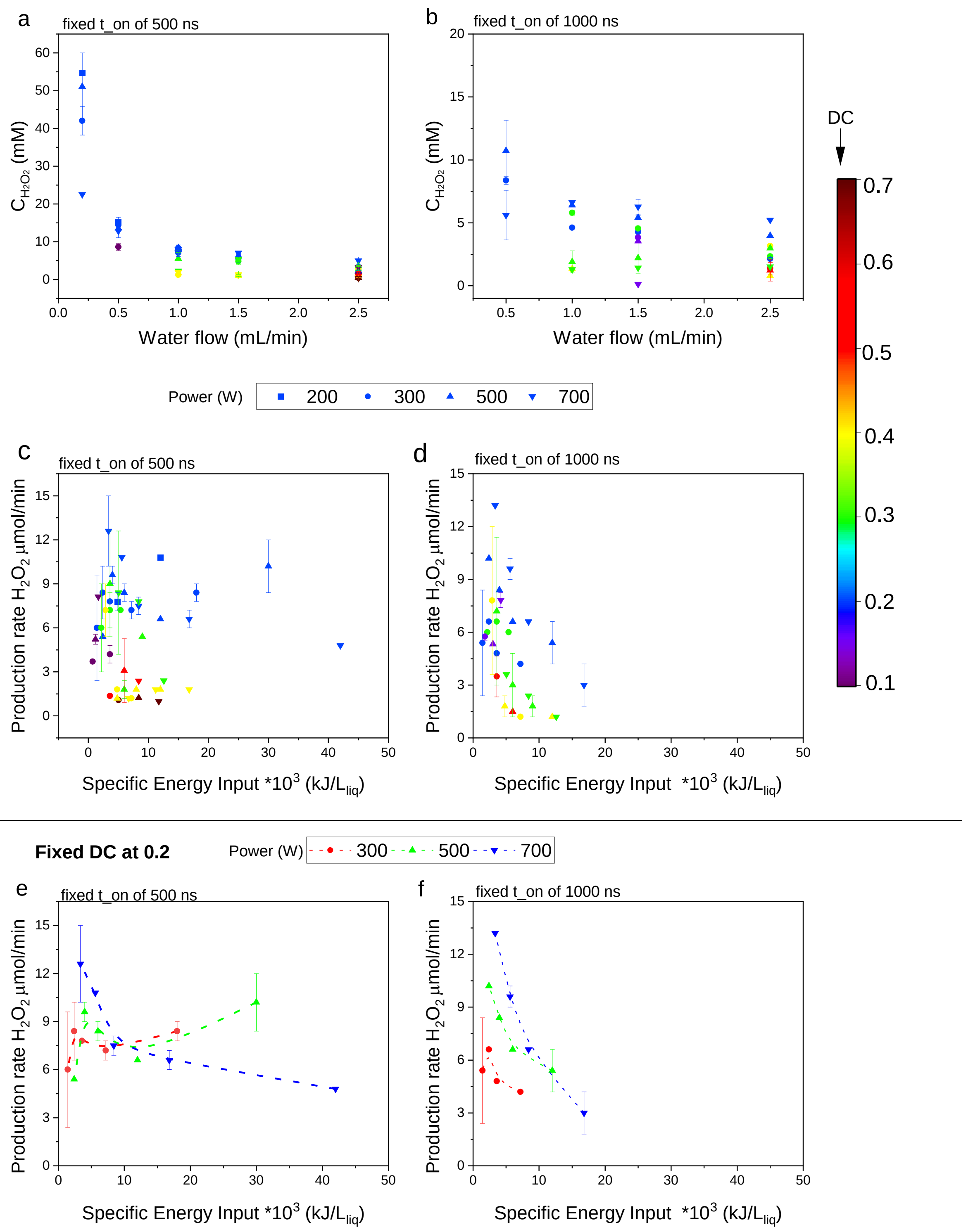

Figure S7. Water flow variation over the H_2O_2 concentration and production rate. A) H_2O_2 concentration variation along water flow rate at 500 ns of pulsation time and DC variation, B) H_2O_2 concentration variation along water flow rate at 1000 ns of pulsation time and DC variation, C) H_2O_2 production rate variation along SEI at 500 ns of pulsation time and DC variation, D) H_2O_2 production rate variation along SEI at 1000 ns of pulsation time and DC variation, E) H_2O_2 production rate variation along SEI at 500 ns of pulsation time at 0.2 DC, F) H_2O_2 production rate variation along SEI at 1000 ns of pulsation time at 0.2 DC. The argon flow rate was fixed to 8.7 L/min, while varying the peak power at 200 W (square), 300 W (circle), 500 W (up-triangle) and 700 W (down-triangle), and the duty cycle (DC) from 0.1 to 0.7, which is represented by color mapping. The error bars in the figures include three replicates.

Figures S7A and 7B present the H_2O_2 concentration as a function of the water flow rate variation for 500 ns and 1000 ns of pulsation time, respectively. The variation of such parameters had a lower impact over the H_2O_2 concentration, in comparison to the water flow rate variation (Fig. S7A-B). Also, after changing the time of pulsations from 500 ns to 100 ns, the more influencing factor over the H_2O_2

concentration is still the water flow rate (Fig. S7B). Furthermore, the production rate as a function of the specific energy input from (in terms of the liquid flow), is presented in Figure S7C-D, for both pulsation times respectively. A clear trend is hard to visualize, however, the highest production rates were achieved at around $5x10^3$ kJ/L(liquid). This means lower specific energy inputs lead to higher production rates. Interestingly, not always lower flow rates correlate with lower production rates. For example, when using the lowest flow rate of 0.2 mL/min, which correlates with the highest values of SEI_L (>$30x10^3$ kJ/L(liquid)), it is possible to achieve also higher production rates, given the higher H_2O_2 concentrations obtained (Fig. S7D). A zoom into the zone of 0.2 duty cycle (where the best output concentrations and production rates where achieved), at time of pulsations of 500 ns and 1000 ns is presented in Fig. S7E-F. From these curves, the trends at 300 W and 500 W at 500 ns (Fig S7E) appear to be similar, with maximums around SEI < $5x10^3$ kJ/L. These trends seem to differ from the trend at 700 W, where higher SEI turned out in a reduction of the production rate. Similar to the latter, in the case of 1000 ns (Fig. S7F), all trends for the different testes input powers appear to show that lower higher SEI reduces the production rate.

To support the trend observed previously at 2.5 mL/min, but at lower water flow rate, a scan of pulse time has been made using 0.2 mL/min, at three different peak powers (Figure S8). For such flow, the pulse time scan was rather complicated at peak powers below 300 W, due to higher instability of the plasma flame. In fact, at such peak power and flow rate, pulse time values lower than 500 ns were not possible to acquire. However, we showed that at 200 W (Figure 2c in the main text), concentrations peaked also around 50 mM, which is the same range of the peak concentrations observed in Figure S8. In this case, the pulsation frequency for higher concentrations ranged between 0.25 MHz and 0.4 MHz. The insert in the figure presents higher pulse time values of 3000 ns and 5000 ns. For the latter, a decrease in H_2O_2 concentration was observed, similarly to previous results (Figure 2c in the main text). This detailed evaluation at a lower water flow rate serves as proof-of concept and further experimental determination at different flow rates below 1 mL/min are intended for future works.

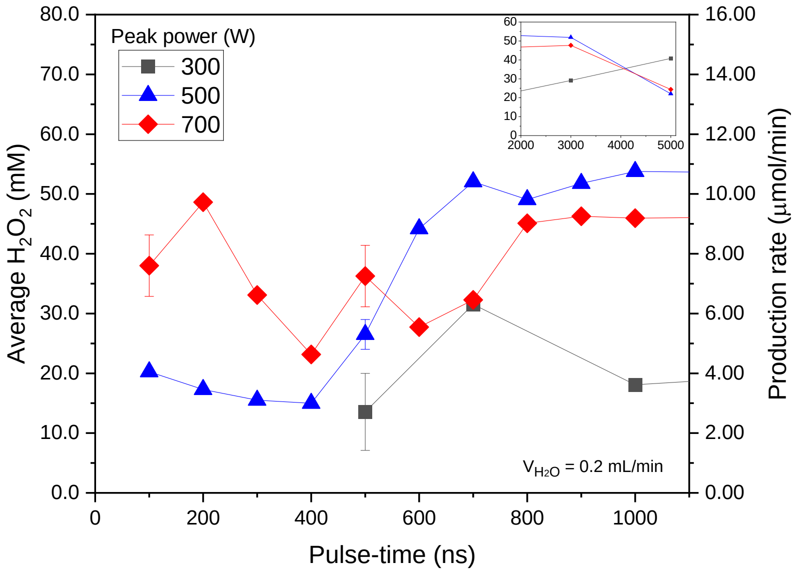

Figure S8. Pulse-time variation over H_2O_2 concentration at 0.2 mL/min. A constant water flow of 0.2 mL/min and a duty cycle of 0.2 were implemented. The peak power was varied at 300, 500 and 700 W.

The energy cost was calculated in grams of produced H_2O_2 per input energy per hour kWh, using the theoretical input energy calculation of peak power multiplied by the duty cycle. Figure S9 summarizes the energy yield along specific energy input variation for different duty cycles. The highlighted trend shows the modulation of the SEI by varying the input power at a constant water flow of 0.2 mL/min. Specifically at 200 W, the SEI rounded the 12 $MJ/L_{(liq)}$ and a maximum of energy yield of 0.56 g/kWh. Other outstanding energy yields at this water flow condition was 0.21 g/kWh at 500 W. However, this does not provide information on the real use of microwave energy. In order to evaluate the effective energy used, the absorbed power was measured at such water flow of 0.2 mL/min and 500 W, following a pulsation time scan (Figure S10). Here, the observed highest energy yield is of 3.7 g/kWh (Figure S10B), which is around 18 times higher than the theoretical value of 0.21 g/kWh at the same conditions. This means that the system is using much less energy than the actual energy input (peak power), and a high percentage (almost 80%) of the energy is reflected or lost.

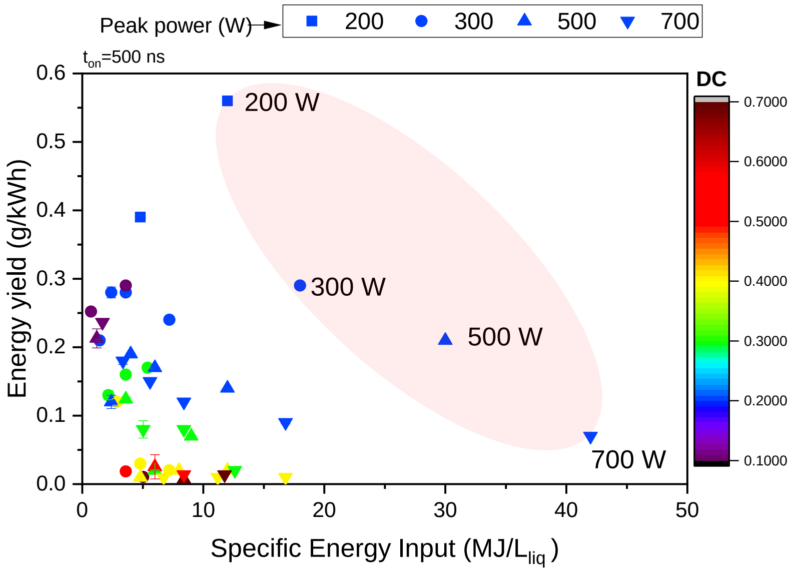

Figure S9. Energy yield variation along Specific energy input. The data was collected using the sepcific case of 500 ns of pulsation time and a scan of duty cycle.

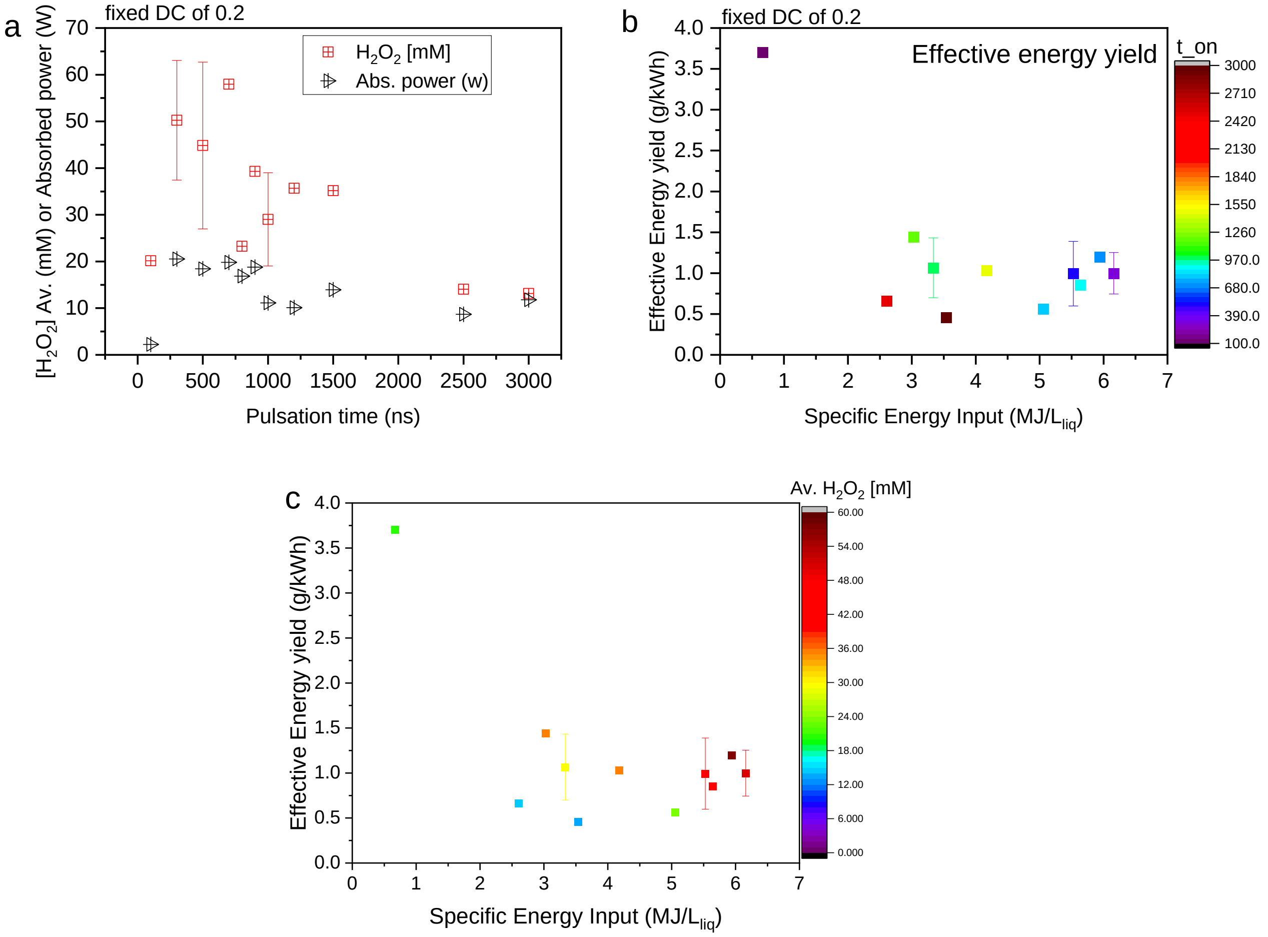

Figure S10. Measurements of absorbed power along pulsation time to correlate: A) the concentration of H_2O_2, and effective energy yield and specific energy input while vaying pulse time B) and H_2O_2 concentration C). This results present the evaluation of the energy yield and specific energy input according to the measured absorbed power at 0.2 DC, 500 W of average input power, 0.2 mL/min of water flow and a scan of pulsation time.

The analysis of the energy usage and energy yield serves as basis to improve the energy efficiency of the system. Mainly, a modification of the applied energy must be implemented to avoid high energy losses.

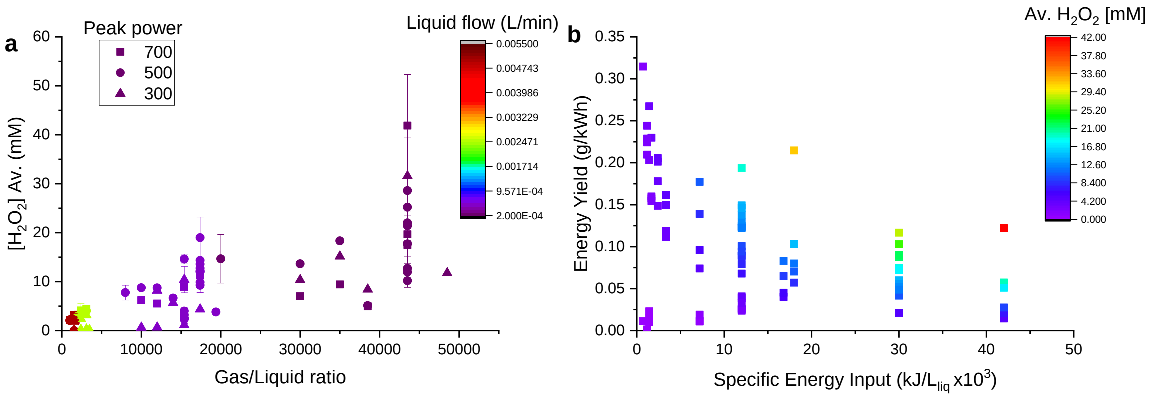

Figure S11. Influence of gas to liquid ratio on H_2O_2 at different peak powers. (a) H_2O_2 concentration (left graphic) and (b) Energy yield versus specific energy input (right graphic). Gas flow rates were varied between 4 and 10 L/min, represented alongside water flow rates shown in the color map. The duty cycle was fixed at 0.2 and pulse at 500 ns.

Tha variation of gas to liquid ratio is presented in Figure S11. From this data, we established that 4 L/min is the minimum gas flow rate required to sustain continuous plasma-water interaction and maintain an active plasma flame in this configuration. The results demonstrate a general trend where higher gas-to-liquid ratios correspond to higher H_2O_2 concentrations across all tested water flow rates. The maximum ratio tested was approximately $48x10^3$, which, at a power input of 300 W, resulted in H_2O_2 concentrations of around 10 mM. However, at higher power inputs, this ratio primarily caused water evaporation, complicating the recovery of liquid samples.

Mass balance

The following global reaction was assumed to calculate the mass balance was:

$$a\, H_2O \rightarrow bH_2O_2 + cH_2 + dO_2$$

To obtain the coefficients, we calculated the amount of water steam using the global mass balance as follows:

$$W_{H2O(l),\, in} = W_{H2O(l),out} + W_{H2O2\, out} + W_{H2,\, out} + W_{H2O(s),\, out} + W_{O2\, out}$$

Where the mass of each component is represented by W and the subindex clarifies whether it is a product (out) or a reactant (in).

Then the steam quantity can be obtained from:

$$W_{H2O(s),\, out} = W_{H2O(l),in} - \left(W_{H2O(l),\, out} + W_{H2O2\, out} + W_{H2,\, out} + W_{O2,out}\right)$$

The balance was checked using a component balance from the global reaction as follows:

$$H: a*2-(b*2+c*2) = 0$$

$$O: a - (b*2+d*2) = 0$$

The reacted quantity of H_2O can be calculated as:

$$H_2O_{reacted} = H_2O_{(l),\, in} - H_2O_{(l),\, out} - H_2O_{(steam),\, out}$$

Example of balance calculations using one case, at 700 W of input power with a DC of 0.3, where the gas composition was:

Gas	Vol%
H_2	$6.2x10^{-4}$
O_2	$3x10^{-4}$

The total gas flow was 8.7 L/min, with a sampling time of 1.5092 min. The total volume of gas during the sample was then calculated as 13.1283 L. To find the total gas moles:

$$13.13\ L * \frac{1\ mol}{22.4}L = 0.5861\ mol\ of\ gas\ total$$

The liquid flow rate was 2.5 mL/min. Then, the total volume of water fed was:

$$H_2O_{in} = 2.5\ mL/\min * 1.5092\min = 3.773\ mL = 0.2096\ mol$$

Mole and Mass Balance

- **Gas phase**

H_2 formed: $6.2x10^{-4} * 0.5861 = 3.6341x10^{-4}mol = 7.2664x10^{-4}g$

O_2 formed: $3x10^{-4} * 0.5861 = 1.7584x10^{-4}\ mol = 56.256x10^{-4}g$

- **Liquid Phase**

Sample weight (W_S): $3.3878\ g \approx 3.3878$ mL

H_2O_2 concentration: $4.66\ mM = 4.66x10^{-3}mol/L$

$n_{H_2O_2}$: $\frac{4.66x10^{-3}mol}{L} * 3.3878x10^{-3}L = 0.1578x10^{-4}mol$

$W_{H_2O_2}$: $0.1578x10^{-4}mol * \frac{34g}{mol} = 5.3652x10^{-4}g$

$$H_2O_{out} = W_s - W_{H_2O_2} = 3.3878\ g - 5.3652x10^{-4}g = 3.3872\ g = 0.1881\ mol$$

- **Steam**

$$W_{H2O(s),\,out} = W_{H2O(l),\,in} - (W_{H2O(l),\,out} + W_{H2O2\ out} + W_{H2,\,out} + W_{O2,out})$$
$$= 3.773g - (3.3872g + 5.3652x10^{-4}g + 7.2664x10^{-4}g + 56.256x10^{-4}g) = 0.3789\ g$$
$$= 0.02105\ mol$$

- **Reacted water**

$$H_2O_{reacted} = H_2O_{(l),\,in} - H_2O_{(l),\,out} - H_2O_{(steam),\,out}$$

$$H_2O_{reacted} = (3.773 - 3.387 - 0.379) * \frac{1mol}{18\ g}H_2O = 3.828x10^{-4}mol$$

- **Summary**

$$aH_2O \rightarrow bH_2 + cO_2 + dH_2O_2$$

If we normalized all species according to the reacted water amount, we can find the coefficients for the reaction in Table 1:

Table S1. Summary of reactant and product components and their approximated molar coefficients

Species	moles	ratio	Approximation	Average*	Error*
H_2O	$3.828x10^{-4}$	1	1	1	0
H_2	$3.6332x10^{-4}$	0.95	1	1.04	0.2
O_2	$1.758x10^{-4}$	0.46	½	0.45	0.02
H_2O_2	$0.1578x10^{-4}$	0.04	1/25	0.04	0.01

*Average and error (standard deviation) of three repetitions

In this case, the total balance spotted the following coefficients:

$$H_2O \rightarrow H_2 + \frac{1}{2}O_2 + \frac{1}{25}H_2O_2$$

Among the main observations are: 1) a deviation of 0.2 was observed for H_2, 2) the first part of the reaction balance corresponds to water splitting, but adding the generation of H_2O_2 leads a balance inconsistence, and 3) therefore an initial feed of 1 mol of H_2O would not represent an accurate stoichiometric balance for producing 1/25 H_2O_2. Considering potential human error and the variation in composition across different tests, a recalculation of the coefficients led to a tentative proposed reaction:

$$27\ H_2O \rightarrow 26\ H_2 + \frac{25}{2}\ O_2 + H_2O_2$$

The component balance, selectivity and products are presented in Tables S1 and S2.

For the gas analysis, an ABB AO2000 continuous gas analyser was utilized, equipped with three advanced detection modules for specific gases.(4) The Uras module, based on infrared analysis, facilitated the detection of CO_2, CO, and CH_4 with a sensitivity threshold of <0.4%. The Caldos module, employing thermal conductivity analysis, enabled the detection of H_2 with a detection limit of <1%. Additionally, the Magnos module, designed for oxygen analysis, achieved a remarkable detection limit of <25 ppm. For monitoring sample weight, a highly precise scale with a detection accuracy up to four decimal places was used.

Tables S2 and S3. Mass balance for H_2O_2 plasma synthesis. The H_2 and O_2 concentrations were followed using an on-line gas analyzer AO2000 (ABB). The microwave power was varied between 300, 500 and 700 W, for a water flow of 2.5 mL/min. The pulsation time was set to 500 ns and the DC was varied between 0.2 and 0.3. The component balance for H and O, for experiments without quenching, is shown in Table S2, and for experiments with quenching in Table S3.

Table S2. Component balance – Tests without quenching

	DC	SEI	Selectivity						Products (g/min)				Elementary balance	
	-	kJ/L_{H2O}	H_2O_2	δ	H_2	δ	O_2	δ	H_2O_2	H_2	O_2	H_2O steam	H	O
300	0.2	1440	0.0296	0.0022	0.6308	0.0604	0.3396	0.0621	0.0003	0.0003	0.0028	0.2661	0.147	-0.007
500	0.2	2400	0.0221	0.0028	0.5685	0.0556	0.4095	0.0582	0.0003	0.0005	0.0053	0.3286	0.560	-0.033
700	0.2	3360	0.0299	0.0016	0.6334	0.0367	0.3368	0.0382	0.0004	0.0005	0.0039	0.0778	0.153	-0.013
300	0.3	2160	0.0345	0.0069	0.6497	0.0673	0.3158	0.0742	0.0003	0.0003	0.0026	0.2961	-0.020	0.007
500	0.3	3600	0.0273	0.0049	0.6583	0.0201	0.3144	0.0241	0.0004	0.0005	0.0039	0.2618	-0.013	0.000
700	0.3	5040	0.0288	0.0019	0.6742	0.0452	0.2971	0.0470	0.0004	0.0005	0.0036	0.2902	-0.167	0.007

Table S3. Component balance – Tests with quenching

Power	DC	SEI	Selectivity						Products (g/min)				Elementary balance	
W	-	kJ/L_{H2O}	H_2O_2	δ	H_2	δ	O_2	δ	H_2O_2	H_2	O_2	H_2O steam	H	O
300	0.2	1440	0.0302	0.0113	0.7232	0.0399	0.2466	0.0511	0.0003	0.0004	0.0024	0.1133	-0.650	0.040
500	0.2	2400	0.0261	0.0078	0.6219	0.1860	0.3520	0.1919	0.0004	0.0005	0.0056	0.1408	-0.073	0.007
700	0.2	3360	0.0242	0.0066	0.6395	0.1481	0.3363	0.1525	0.0004	0.0006	0.0058	0.2006	-0.087	0.013
300	0.3	2160	0.0230	0.0030	0.6423	0.1044	0.3347	0.1018	0.0002	0.0004	0.0034	0.2514	0.013	0.000
500	0.3	3600	0.0160	0.0043	0.6482	0.1086	0.3358	0.1043	0.0002	0.0005	0.0043	0.1032	-0.020	0.013
700	0.3	5040	0.0062	0.0025	0.6272	0.1045	0.3666	0.1057	0.0001	0.0005	0.0050	0.1645	0.147	-0.013

From our observations, slightly higher steam was produced for most of the tests without cooling than with cooling (Fig. S12). This is consistent with reaching higher temperatures in the plasma-water interaction zone when no quenching is implemented. On the other hand, the H_2O_2 production rate seems to be more predictable when employing a lower DC of 0.2 and cooling, as marked in dotted line (guide for the eye) in Figure S12. Without cooling, there was not a clear relation between steam generation and H_2O_2 production, and it was rather more difficult to control the H_2O_2 production. The direct correlation with steam is not the focus of this paper and remains a matter of further research.

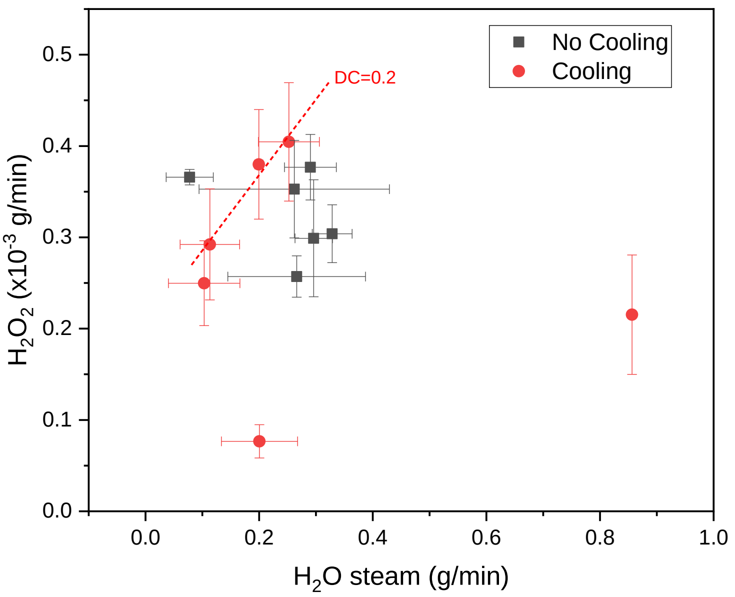

Figure S12. Steam generation influence on hydrogen peroxide production. Experiments were carried out without and with cooling. The microwave power varied between 300, 500 and 700 W, for a water flow rate of 2.5 mL/min. The pulse time was set to 500 ns and the DC varied between 0.2 and 0.3.

Section 3: High resolution time-resolved spectroscopy

Nanosecond time resolved Optical Emission Spectroscopy (OES)

The spectrometer includes high-resolution spectrograph Acton SP-2756 with 750 mm focal length and intensified (ICCD) CCD camera from Andor, (type A-DH340-18U-03), with a chip size of 512×2048 pixels and a pixel size of 13.5 μm. The spectrometer was calibrated in wavelength as well as in signal intensity together with the ICCD camera, the lens and fiber optic cable. For the wavelength calibration and for relative intensity calibration, the mercury lamp (from Princeton Instruments) and the USB-LSVN VIS-NIR lamp (from Princeton Instruments) were used, respectively. The light emitted by plasma discharge is collected with SMA focusing lens and guided by multi-fiber cable to the entrance slit of the spectrograph. In the present work, a holographic type grating with 1800 lines per mm was utilized. The ICCD camera features a fast gating time (>2 ns) thus enabling nanosecond time resolution. The spectral resolution was 0.1 nm. To follow the spectral plasma properties within a single period of microwave pulsation the gate of the ICCD camera is synchronized with the microwave pulses. For that purpose, the pulse-form output TTL signal from microwave source is used as a trigger for ICCD camera. Time steps are realized by setting the corresponding time delay (accuracy is better than 10 ps) which is realized with the digital delay generator (SRS DG 645) installed between ICCD and microwave source (see Figure S13). The axis of collecting lens was inclined with respect to plasma torch axis in such a way that it enables the optimum line of sight aimed to plasma-water interface.

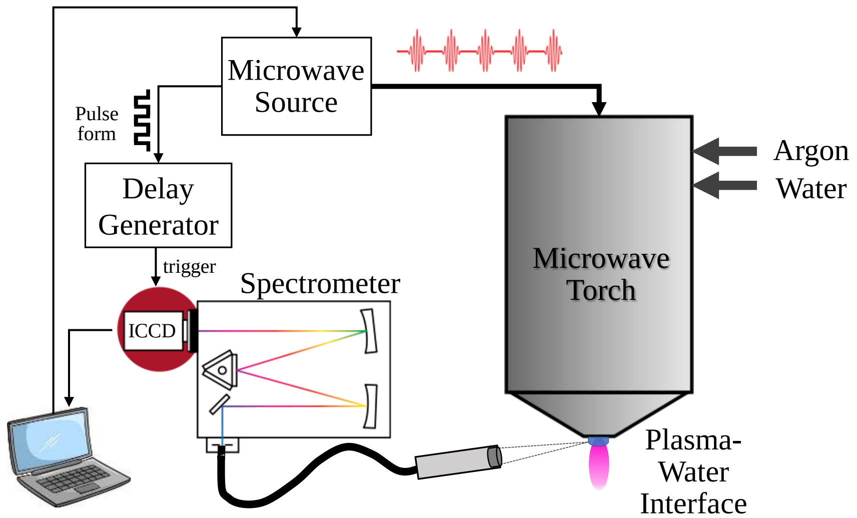

Figure S13. The scheme of acquisition of emitted light from plasma. ICCD camera gate is synchronized with microwave pulsation and enabling the time resolved spectra acquisition. Time steps are realized through the corresponding time delay in the trigger signal.

The time-resolved high-resolution spectra of the Hβ line allows estimate the electron density (Ne). Ne can be calculated from the Hβ line using with equation 1. (5)

Equation 1:

$$FWHM = 4.800\ nm\ x \left(\frac{N_e}{10^{23}\,m^{-3}}\right)^{0.68116}$$

$$n_e = \left(\frac{FWHM}{4.800\ nm}\right)^{1.46808} x\,10^{23}\,m^{-3}$$

FWHM: Full width at half maximum

Section 4: Plasma–liquid reactions analysis and system performance

Table S4. Overview of reactions classified according to the phase where they are likely to occur during plasma–liquid interactions. The table includes the rate and temperature found in literature, and the estimated enthalpy.

**Plasma-liquid Interface**	**Rate constant *k***	**TEMPERATURE**	**Reference**	**ΔH (eV/mol)***
$H_2O + e^- \rightarrow OH + H + e^-$	$2.3x18^{-18}$ – $1.8 x10^{-16}$ (m^3s^{-1})	Te = 1-2 eV	(9)	5.17
$H_2O + M \rightarrow OH + H + M$	$5.8x10^{-9}$exp(-440000(RT)^-1)	Tg =2-6x10^3K	(10)	
$H_2O + Ar^* \rightarrow e^- + H_2O^+ + Ar$			(9)	19.63
$H_2O^+ + e^- \rightarrow OH + H$			(9,11,12)	-14,46
$H_2O + Ar^+ \rightarrow OH + H + Ar$	$4.5x10^{-16}$ (m^3s^{-1})	Tg = 300 K	(13)	-10.58
$H_2O + H_2O \rightarrow OH + H + H_2O$	10^{-26} – $4x10^{-20}$ (m^3s^{-1})	Tg=2500-5000 K	(13)	
$H_2O_{(l)} + H_2O^* \rightarrow OH + H + H_2O$	$x10^{-20}$ (m^3s^{-1})	Tv=0.5 eV, Tg=300 K	(13)	4.82
$OH + H_2O^* \rightarrow H_2O_2 + H$			(8)	2.6^+
$H + H_2O^* \rightarrow H_2 + OH$			(8)	0.6^+
$H_2O^+ + H_2O \rightarrow OH^- + H_3O^+$	$1.9x10^{-15}m^3s^{-1}$		(13–15)	-19.51
**Gas phase**				
$HO_2 + H_2O^* \rightarrow H_2O_2 + OH$			(8)	1.1^+
$OH + H_2O^* \rightarrow H_2 + HO_2$			(8)	2.1^+
$OH + OH + M \rightarrow H_2O_2 + M$	$2.6x10^{-17}$(m^3s^{-1})		(13)	-2.22
	$3.7x10^{-43}$(T_g/300)$^{-0.8}$(cm^3/s), when M=He		(16)	
	$1.51x10^{-11}$ (T/298)$^{-0.37}$	300-1500 K	(10)	
$H + O_2 \rightarrow HO_2$	$9.57x10^{-8}cm^3mol^{-2}s^{-1}$		(17)	-2.14
$H + O_2 + M \rightarrow HO_2 + M$	$1.94x10^{-32}$(T/298)$^{-1}$	200-2200K	(10)	
$HO_2 + HO_2 \rightarrow H_2O_2 + O_2$	$3.01x10^{-12}$ s^{-1}	300-2500 K	(10)	-1.66
$OH + HO_2 \rightarrow H_2O + O_2$	$x10^{-11}cm^3mol^{-1}s^{-1}$		(18)	-3.03
	$x10^{10}$ $M^{-1}s^{-1}$		(14)	
	$2.6x10^{-11}$exp(370.85/Tg) (cm^3/s)		(16)	
	$4.81x10^{-11}$exp(-2031000(RT)$^{-1}$)	300-2000K	(10)	
$H + H_2O_2 \rightarrow H_2 + HO_2$	$2.6x10^{-11}$exp$^{(-3162/Tg)}$ (cm^3/s)		(16)	-0.73
	$8x10^{-11}$ exp(-33260 (RT)$^{-1}$	300-2500K	(10)	
$OH + H_2O_2 \rightarrow H_2O + HO_2$	$1x10^{10}$ $M^{-1}s^{-1}$		(14)	-1.38
	$2.88x10^{-12}$ (exp(-156.3/T_g))		(16)	
	$2.91x10^{-12}$ exp(-1330 (RT)$^{-1}$)	300 -2500K	(10)	
$O + H_2O_2 \rightarrow OH + HO_2$	$1.11x10^{-12}$exp(-1943.6/T_g)		(16)	-0.64
**Liquid phase**				
$HO_2^- + H^+ \rightarrow H_2O_2$			(11,12)	-1.29
$H_3O^+ + HO_2^- \rightarrow H_2O_2 + H_2O$	($3x10^{10}$ $M^{-1}s^{-1}$)		(14)	-1.29
$HO_2^- + H_2O_{(l)} \rightarrow H_2O_2 + OH^-$	($1.1x10^{10}$ $M^{-1}s^{-1}$)		(14)	4.06
$OH + O^- \rightarrow HO_2^-$	($2.7x10^{10}$ $M^{-1}s^{-1}$)		(14)	-2.70
$OH + OH^- \rightarrow H_2O + O^-$	($1.2x10^{10}$ $M^{-1}s^{-1}$)		(14)	1.08
	$1.2x10^{-12}$ $cm^3mol^{-1}s^{-1}$		(19)	
	$13x10^9$ $M^{-1}s^{-1}$		(20)	
	$1.3x10^{10}$ $M^{-1}s^{-1}$		(11)	
$e^- + H_2O_2 \rightarrow OH^- + OH$	$11x10^9M^{-1}s^{-1}$		(11)	
$H_2O_2 \rightarrow H_2O + 1/2\ O_2$	$2x10^5$ exp (-14800/RT)		(21)	

*Enthalpy estimated from thermochemical data base. (7)

$^+$Enthalpy reported in the respective reference.

This preliminary reaction scheme is intended as a foundation for future modeling and simulation studies to elucidate the formation pathways of the observed species in this plasma configuration. In this sense, the reactions involving radical-radical interactions typically take place in the gas phase, but the reactions that involve high-energy plasma generated species (electrons, excited water, ionized argon) interacting with liquid water molecules, take place most likely in the plasma-liquid interface; and ultimately, the reactions involving ions (OH^-, H_3O^+, HO_2^-) interacting in solution, indicate they occur in the bulk liquid phase. OH ions are mostly formed in the liquid phase due to lower energy requirements for their life.

Residence time and PFR reactor model

The residence time (Figure S14) was evaluated for a scan of water flow rates (0.2 mL/min – 2.5 mL/min) at peak powers of 200W, 300W, 500W and 700W, at a constant DC of 0.2 and pulse time of 500 ns. Using a simple plug flow reactor model one can follow the net reaction rate by subtracting the formation rate from the decomposition rate. At the same time, we discussed that, depending on the power (at fixed pulsation conditions) the decomposition or decay can change, with higher powers leading to more decomposition. Thus, one can propose the following equation for each power level:

$$r_{Net} = r_f - r_d$$

Considering a liquid plug-flow element with constant formation and first-order decay we obtain:

$$\frac{dC_{HP}}{dt} = r_f - k_d C_{HP}$$

With $C_{HP}(0) = 0$ as initial condition.

Considering residence time, the solution to this first order, linear differential equation is:

$$C_{HP}(\tau) = \frac{r_f}{k_d}\left(1 - e^{-k_d \tau}\right)$$

Which considering the Damköhler number for first-order reactions can also be written as:

$$C_{HP}(Da_d) = \frac{r_f}{k_d}\left(1 - e^{-Da_d}\right)$$

Table S6. Results of PFR model to follow the formation and decomposition rate following the given experimental conditions in Figure S14.

Power (W)	Formation rate constant k (M s^{-1})	Decomposition rate constant k (1/s)
200	0.00809	0
300	0.00584	0
500	0.00748	0
700	0.00318	0.04

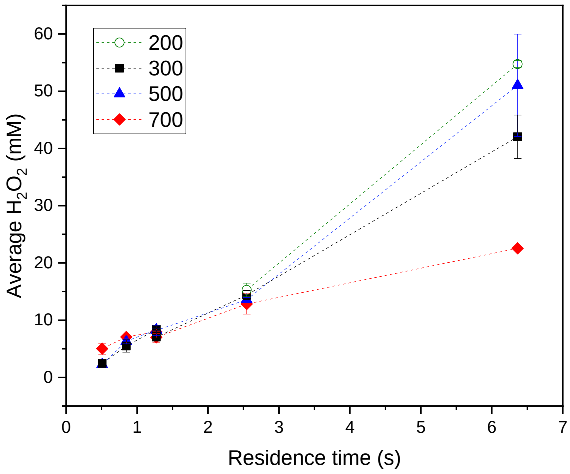

Figure S14. Effect of residence time on the produced H_2O_2 concentration at different peak microwave power, at a fixed DC of 0.2 and pulse time of 500 ns. Constant plasma volume assumed of 0.021 mL.

Benchmarking

The system is highly comparable with other reports where only water and plasma are employed for H_2O_2 production. In Figure S15, we highlight these contributions and compare the achieved H_2O_2 concentration and energy yield (left y-axis) as well as the production rate (right y-axis), including the best scenario from this work. For context, we also included an example of an electrocatalytic system, in which the use of a boron-doped diamond anode significantly enhances H_2O_2 yield.

To simplify reference tracking, a table is provided below with the corresponding literature sources listed in alphabetical order.

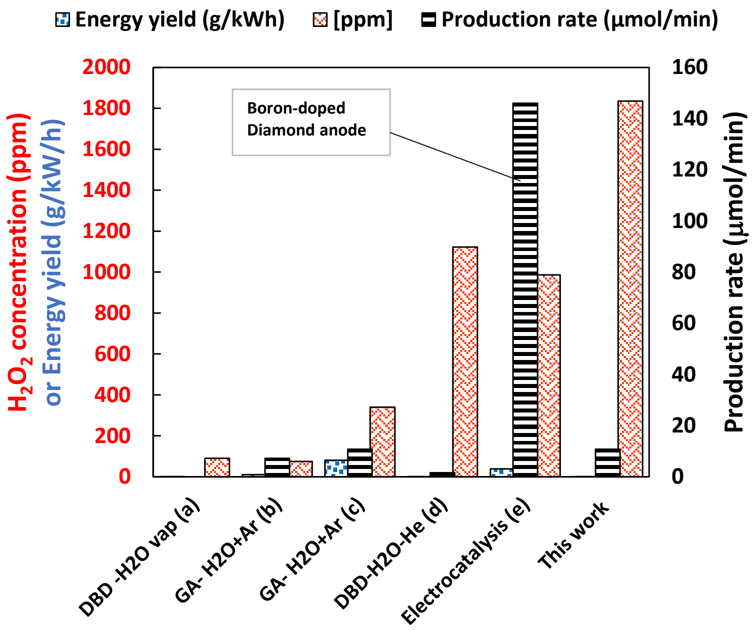

Figure S15. Comparison of H_2O_2 concentration, production rate and energy yield among different plasma systems. The best-case scenario from this work is included, as well as an example of electrochemical synthesis (reference "e").

References for Figure S15:

a	Tachibana, K., & Nakamura, T. (2019). Japanese Journal of Applied Physics, 58(4), 046001.
b	Wandell, R. J., & Locke, B. R. (2014). Industrial & Engineering Chemistry Research, 53(2), 609-618.
c	Burlica, R., Shih, K. Y., & Locke, B. R. (2010). Industrial & Engineering Chemistry Research, 49(14), 6342-6349.
d	Cameli, F., Dimitrakellis, P., Chen, T. Y., & Vlachos, D. G. (2022). ACS Sustainable Chemistry & Engineering, 10(5), 1829-1838.
e	Mavrikis, Sotirios, et al. ACS Applied Energy Materials 3.4 (2020): 3169-3173.

Stability along time

Long-term experiments (~6.5 hours) were conducted to investigate the continuous production of H_2O_2. The variations in product concentration over time (Figure S16) are attributed to the dynamic nature of the plasma–water interaction within this reactor, which fluctuates with the intermittent water droplet feed and the pulsed microwave operation. The error bars represent the standard deviation of three measurements performed on different days.

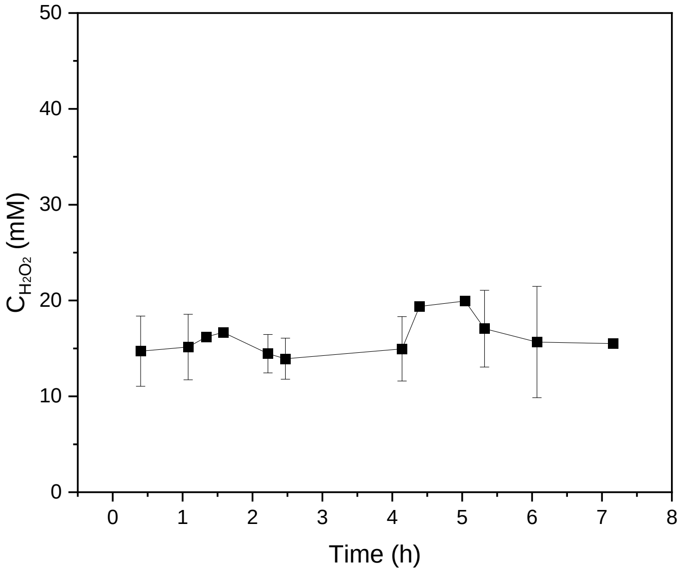

Figure S16. Long-term experiments of plasma driven H_2O_2 production. The tests were made using a water flow rate of 0.5 mL/min, a gas flow rate of 8.7 L/min, a fixed pulse time of 500 ns and DC of 0.2, with a peak power of 500 W.

References

(1) NASA Glenn Research Center. *"Chemical Equilibrium with Applications (CEA)."* https://cearun.grc.nasa.gov/ (accessed 2024-08-28).

(2) Eisenberg, G. M. *Colorimetric Determination of Hydrogen Peroxide Optimum Quantity of Test Reagent for Maximum Color Development*. https://pubs.acs.org/sharingguidelines.

(3) Satterfield, C. N.; Bonnell, A. H. *Interferences in the Titanium Sulfate Method for Hydrogen Peroxide*; UTC; Vol. 56. https://pubs.acs.org/sharingguidelines.

(4) ABB Library. Advance Optima Continuous Gas Analyzers AO2000 Series.

(5) Bruggeman, P.; Schram, D.; González, M. Á.; Rego, R.; Kong, M. G.; Leys, C. Characterization of a Direct Dc-Excited Discharge in Water by Optical Emission Spectroscopy. *Plasma Sources Sci. Technol.* **2009**, *18*. https://doi.org/10.1088/0963-0252/18/2/025017.

(6) Ruscic, B.; Pinzon, R. E.; Morton, M. L.; Von Laszevski, G.; Bittner, S. J.; Nijsure, S. G.; Amin, K. A.; Minkoff, M.; Wagner, A. F. *Introduction to active thermochemical tables: Several "Key" enthalpies of formation revisited*. Journal of Physical Chemistry A. https://doi.org/10.1021/jp047912y.

(7) Atkins, P.; de Paula, J. *Atkins' Physical Chemistry*; 2010; Vol. 11.

(8) Fridman, A. A. . *Plasma Chemistry*; Cambridge University Press. https://doi.org/https://assets.cambridge.org/97805218/47353/frontmatter/9780521847353_frontmatter.pdf.

(9) Qazi, H. I. A.; Nie, Q. Y.; Li, H. P.; Zhang, X. F.; Bao, C. Y. Comparison of Electrical and Optical Characteristics in Gas-Phase and Gas-Liquid Phase Discharges. *Phys. Plasmas* **2015**, *22* (12). https://doi.org/10.1063/1.4937779.

(10) Chauvet, L.; Nenbangkaeo, C.; Grosse, K.; von Keudell, A. Chemistry in Nanosecond Plasmas in Water. *Plasma Process. Polym.* **2020**, *17* (6). https://doi.org/10.1002/ppap.201900192.

(11) Chen, Q.; Li, J.; Chen, Q.; Ostrikov, K. Recent Advances towards Aqueous Hydrogen Peroxide Formation in a Direct Current Plasma–Liquid System. *High Voltage*. John Wiley and Sons Inc June 1, 2022, pp 405–419. https://doi.org/10.1049/hve2.12189.

(12) Locke, B. R.; Shih, K. Y. Review of the Methods to Form Hydrogen Peroxide in Electrical Discharge Plasma with Liquid Water. *Plasma Sources Science and Technology*. June 2011. https://doi.org/10.1088/0963-0252/20/3/034006.

(13) Bruggeman, P.; Schram, D. C. On OH Production in Water Containing Atmospheric Pressure Plasmas. *Plasma Sources Sci. Technol.* **2010**, *19* (4). https://doi.org/10.1088/0963-0252/19/4/045025.

(14) Oinuma, G.; Nayak, G.; Du, Y.; Bruggeman, P. J. Controlled Plasma-Droplet Interactions: A Quantitative Study of OH Transfer in Plasma-Liquid Interaction. *Plasma Sources Sci. Technol.* **2020**, *29*. https://doi.org/10.1088/1361-6595/aba988.

(15) Lietz, A. M.; Kushner, M. J. Air Plasma Treatment of Liquid Covered Tissue: Long Timescale Chemistry. *J. Phys. D. Appl. Phys.* **2016**, *49*. https://doi.org/10.1088/0022-3727/49/42/425204.

(16) Vasko, C. A.; Liu, D. X.; Van Veldhuizen, E. M.; Iza, F.; Bruggeman, P. J. Hydrogen Peroxide Production in an Atmospheric Pressure RF Glow Discharge: Comparison of Models and Experiments. *Plasma Chem. Plasma Process.* **2014**, *34* (5), 1081–1099. https://doi.org/10.1007/s11090-014-9559-8.

(17) Elliot, A. J. Rate Constants And G-Values For The Simulation Of The Radiolysis Of Light Water Over The Range 0-300°C. *Atomic Energy of Canada Limited*. 1994, pp 1–69.

(18) Keyser, L. F. Kinetics of the Reaction OH + HO2 → H2O + O2 from 254 to 382 K. *J. Phys. Chem.* **1988**, *92* (5), 1193–1200. https://doi.org/10.1021/J100316A037/ASSET/J100316A037.FP.PNG_V03.

(19) Sun, Q. The Raman OH Stretching Bands of Liquid Water. *Vib. Spectrosc.* **2009**, *51* (2), 213–217. https://doi.org/10.1016/j.vibspec.2009.05.002.

(20) Lin, J.; He, X.; Chen, Q.; Xiong, Q.; Li, J.; Wang, X.; Chen, G.; Liu, Q. H.; Ostrikov, K. (Ken). The Formation Mechanism of Aqueous Hydrogen Peroxide in a Plasma-Liquid System with Liquid as the Anode. *Eur. Phys. J. D* **2020**, *74* (4). https://doi.org/10.1140/epjd/e2020-100371-2.

(21) Lin, C. C.; Smith, F. R.; Ichikawa, N.; Baba, T.; Itow, M. Decomposition of Hydrogen Peroxide in Aqueous Solutions at Elevated Temperatures. *Int. J. Chem. Kinet.* **1991**, *23* (11), 971–987. https://doi.org/https://doi.org/10.1002/kin.550231103.
